# Likelihood analysis of supersymmetric SU(5) GUTs

**DOI:** 10.1140/epjc/s10052-017-4639-6

**Published:** 2017-02-16

**Authors:** E. Bagnaschi, J. C. Costa, K. Sakurai, M. Borsato, O. Buchmueller, R. Cavanaugh, V. Chobanova, M. Citron, A. De Roeck, M. J. Dolan, J. R. Ellis, H. Flächer, S. Heinemeyer, G. Isidori, M. Lucio, D. Martínez Santos, K. A. Olive, A. Richards, K. J. de Vries, G. Weiglein

**Affiliations:** 1 DESY, Notkestraße 85, 22607 Hamburg, Germany; 2 High Energy Physics Group, Blackett Laboratory, Imperial College, Prince Consort Road, London, SW7 2AZ UK; 3 Department of Physics, Institute for Particle Physics Phenomenology, University of Durham, Science Laboratories, South Road, Durham, DH1 3LE UK; 4 Faculty of Physics, Institute of Theoretical Physics, University of Warsaw, ul. Pasteura 5, 02-093 Warsaw, Poland; 5 Universidade de Santiago de Compostela, 15706 Santiago de Compostela, Spain; 6 Fermi National Accelerator Laboratory, P.O. Box 500, Batavia, IL 60510 USA; 7 Physics Department, University of Illinois at Chicago, Chicago, IL 60607-7059 USA; 8 Experimental Physics Department, CERN, 1211 Geneva 23, Switzerland; 9 Antwerp University, 2610 Wilrijk, Belgium; 10 ARC Centre of Excellence for Particle Physics at the Terascale, School of Physics, University of Melbourne, Parkville, 3010 Australia; 11 Theoretical Particle Physics and Cosmology Group, Department of Physics, King’s College London, London, WC2R 2LS UK; 12 Theoretical Physics Department, CERN, 1211 Geneva 23, Switzerland; 13 H.H. Wills Physics Laboratory, University of Bristol, Tyndall Avenue, Bristol, BS8 1TL UK; 14 Campus of International Excellence UAM+CSIC, Cantoblanco, 28049 Madrid, Spain; 15 Instituto de Física Teórica UAM-CSIC, C/Nicolas Cabrera 13-15, 28049 Madrid, Spain; 16 Instituto de Física de Cantabria (CSIC-UC), Avda. de Los Castros s/n, 39005 Santander, Spain; 17 Physik-Institut, Universität Zürich, 8057 Zurich, Switzerland; 18 William I. Fine Theoretical Physics Institute, School of Physics and Astronomy, University of Minnesota, Minneapolis, MN 55455 USA

## Abstract

We perform a likelihood analysis of the constraints from accelerator experiments and astrophysical observations on supersymmetric (SUSY) models with SU(5) boundary conditions on soft SUSY-breaking parameters at the GUT scale. The parameter space of the models studied has seven parameters: a universal gaugino mass $m_{1/2}$, distinct masses for the scalar partners of matter fermions in five- and ten-dimensional representations of SU(5), $m_5$ and $m_{10}$, and for the $\mathbf {5}$ and ${\bar{\mathbf{5}}}$ Higgs representations $m_{H_u}$ and $m_{H_d}$, a universal trilinear soft SUSY-breaking parameter $A_0$, and the ratio of Higgs vevs $\tan \beta$. In addition to previous constraints from direct sparticle searches, low-energy and flavour observables, we incorporate constraints based on preliminary results from 13 TeV LHC searches for jets +  events and long-lived particles, as well as the latest PandaX-II and LUX searches for direct Dark Matter detection. In addition to previously identified mechanisms for bringing the supersymmetric relic density into the range allowed by cosmology, we identify a novel ${\tilde{u}_R}/{\tilde{c}_R} - \tilde{\chi }^{0}_{1}$ coannihilation mechanism that appears in the supersymmetric SU(5) GUT model and discuss the role of ${{\tilde{\nu }}_\tau }$ coannihilation. We find complementarity between the prospects for direct Dark Matter detection and SUSY searches at the LHC.

## Introduction

In the absence so far of any experimental indications of supersymmetry (SUSY) [1–9], nor any clear theoretical guidance how SUSY may be broken, the building of models and the exploration of phenomenological constraints on them  [10–17] have adopted a range of assumptions. One point of view has been to consider the simple parametrization of soft SUSY breaking in which the gaugino and scalar masses, as well as the trilinear soft SUSY-breaking parameters, are all constrained to be universal at the SUSY GUT scale (the CMSSM [10–13, 18–45]). An alternative point of view has been to discard all universality assumptions, and treat the soft SUSY-breaking parameters as all independent phenomenological quantities (the pMSSM  [15, 46–64]), imposing diagonal mass matrices and the minimal flavour violation (MFV) criterion. Intermediate between these extremes, models with one or two non-universal soft SUSY-breaking contributions to Higgs masses (the NUHM1 [10–13, 65–69] and NUHM2 [14, 67–71]) have also been considered.

It is interesting to explore also models that are less simplified than the CMSSM, but not as agnostic as the pMSSM, in that they incorporate a limited number of simplifying assumptions. GUTs motivate the assumption that the gaugino masses are universal, and constraints on flavour-changing neutral interactions suggest that the soft SUSY-breaking masses for scalars with identical quantum numbers are also universal. However, there is no compelling phenomenological reason why the soft SUSY-breaking masses for scalars with different quantum numbers should be universal.

Specific GUTs may also provide some guidance in this respect. For example, in an SO(10) GUT the scalar masses of all particles in a given generation belonging to a single $\mathbf {16}$ representation of SO(10) would be universal, as would those for the $\mathbf {5}$ and ${\bar{\mathbf{5}}}$ SU(5) Higgs representations that belong to a single $\mathbf {10}$ of SO(10) and break electroweak symmetry, as in the NUHM1. In contrast, the SU(5) framework is less restrictive, allowing different masses for scalars in ${\bar{\mathbf{5}}}$ and $\mathbf {10}$ representations [72], and also for the $\mathbf {5}$ and ${\bar{\mathbf{5}}}$ Higgs representations. Thus it is a 1-parameter extension of the NUHM2. In this paper we explore the theoretical, phenomenological, experimental and cosmological constraints on this SU(5)-based SUSY GUT model.

This relaxation of universality is relevant for the evaluation of several different constraints from both the LHC and elsewhere. For example, the most powerful LHC constraints on the CMSSM, NUHM1 and NUHM2 are those from the classic  searches [1–3, 6–8]. These constrain principally the right-handed squarks, whose decays are dominated by the ${\tilde{q}_R} \rightarrow q \,\tilde{\chi }^{0}_{1}$ channel that maximizes the  signature. On the other hand, the decay chains of left-handed squarks are more complicated, typically involving the $\tilde{\chi }^{\pm }_{1}$, resulting in a dilution of the  signature and more importance for final states including leptons. In a SUSY SU(5) GUT, the left-handed squarks and the right-handed up-type squarks appear in $\mathbf {10}$ representations whereas the right-handed down-type squarks appear in ${\bar{\mathbf{5}}}$ representations, with independent soft SUSY-breaking masses. Hence the impacts of the LHC  and other constraints need to be re-evaluated.

The possible difference between the soft SUSY-breaking contributions to the masses of the squarks appearing in a $\mathbf {10}$ of SU(5), i.e., up-type squarks and left-handed down-type squarks, and those appearing in a ${\bar{\mathbf{5}}}$ of SU(5), i.e., right-handed down-type squarks, offers a new avenue for compressing the stop spectrum. Also, as we shall see, with $m_5 \ne m_{10}$ there is the possibility that $m_{{\tilde{u}_R}, {\tilde{c}_R}}$ are much smaller than the other squark masses, leading to another type of compressed spectrum.^1^

In principle, the constraints from flavour observables may also act differently when $m_5 \ne m_{10}$. For example, the soft SUSY-breaking masses of the left- and right-handed charge +2/3 quarks are independent, and flavour observables such as BR($b \rightarrow s \gamma$) and $\mathrm{BR}(B_s \rightarrow \mu ^+\mu ^-)$ depend on both of them, in general.

Another experimental constraint whose interpretation may be affected by the non-universality of scalar masses is $(g-2)_\mu$. A priori, a SUSY explanation of the discrepancy between the Standard Model (SM) prediction and the experimental measurement of $(g-2)_\mu$ requires relatively light smuons, either right- and/or left-handed, which are in $\mathbf {10}$ and ${\bar{\mathbf{5}}}$ representations, respectively. It is interesting to investigate to what extent the tension between a SUSY interpretation of $(g-2)_\mu$ and the LHC constraints on squarks that is present in more constrained SUSY models could be alleviated by the extra degree of freedom afforded by the ${\bar{\mathbf{5}}}$–$\mathbf {10}$ disconnect in SU(5).

Finally, we recall that in large parts of the regions of the CMSSM, NUHM1 and NUHM2 parameter spaces favoured at the 68% CL the relic $\tilde{\chi }^{0}_{1}$ density is brought into the range allowed by Planck [74] and other data via coannihilation with the stau and other sleptons [75–82]. In an SU(5) GUT, the left- and right-handed sleptons are in different representations, ${\bar{\mathbf{5}}}$ and $\mathbf {10}$, respectively. Hence they have different masses, in general, providing more flexibility in the realization of coannihilation. Specifically, as mentioned above, the freedom to have $m_5 \ne m_{10}$ allows the possibility that the right-handed up- and charm-flavour squarks, ${\tilde{u}_R}$ and ${\tilde{c}_R}$, are much lighter than the other squarks, opening up the novel possibility of ${\tilde{u}_R}/{\tilde{c}_R} - \tilde{\chi }^{0}_{1}$ coannihilation, as we discuss below.

Our analysis of the available experimental constraints largely follows those in our previous studies of other variants of the MSSM [10–17], the main new feature being that we incorporate the constraints based on the preliminary results from LHC searches for jets $+$  events with ${\sim } 13$/fb of data at 13 TeV [9]. For this purpose, we recast available results for simplified models with the mass hierarchies $m_{\tilde{g}}\gg m_{\tilde{q}}$ and vice versa. We also include the preliminary constraints from LHC searches in 13-TeV data for the heavy MSSM Higgs bosons and long-lived charged particles, and incorporate in combination the recent PandaX [83] and LUX [84] data.

The SUSY SU(5) GUT model we study is set up in Sect. 2, and our implementations of constraints and analysis procedure are summarized in Sect. 3. Section 4 describes how we characterize different Dark Matter (DM) mechanisms, including the novel ${\tilde{u}_R}/{\tilde{c}_R} - \tilde{\chi }^{0}_{1}$ coannihilation mechanism, ${{\tilde{\nu }}_\tau }$ coannihilation and a hybrid possibility. Section 5 contains our results in several model parameter planes, and Sect. 6 describes various one-dimensional likelihood functions including those for several sparticle masses, $(g-2)_\mu$ and various other observables. Higgs boson branching ratios (BRs) are presented in Sect. 7, followed by a comparison of the SU(5) with the NUHM2 results in Sect. 8. The possibility of a long-lived ${\tilde{\tau }}_1$ is discussed in Sect. 9, and the prospects for direct DM detection are discussed in Sect. 10. Finally, Sect. 11 presents a summary and some conclusions.

## Supersymmetric SU(5) GUT model

We assume a universal, SU(5)-invariant gaugino mass parameter $m_{1/2}$, which is input at the GUT scale, as are the other SUSY-breaking parameters listed below.

We assume the conventional multiplet assignments of matter fields in the minimal superymmetric GUT:

1 $$\begin{aligned} (q_L, u^c_L, e^c_L)_i \in \mathbf {10}_i, \quad (\ell _L, d^c_L)_i \in {\bar{\mathbf{5}}}_i, \end{aligned}$$

where the subscript $i = 1, 2, 3$ is a generation index. The only relevant Yukawa couplings are those of the third generation, particularly that of the *t* quark (and possibly the *b* quark and the $\tau$ lepton) that may play an important role in generating electroweak symmetry breaking. In our discussion of flavour constraints, we assume the MFV scenario in which generation mixing is described by the Cabibbo–Kobayashi–Maskawa (CKM) model. This is motivated by phenomenological constraints on low-energy flavour-changing neutral interactions, as is our assumption that the soft SUSY-breaking scalar masses for the different $\mathbf {10}_i$ and ${\bar{\mathbf{5}}}_i$ representations are universal in generation space, and are denoted by $m_{10}$ and $m_{5}$, respectively. In contrast to the CMSSM, NUHM1 and NUHM2, we allow $m_5 \ne m_{10}$. We assume a universal soft trilinear SUSY-breaking parameter $A_0$.

We assume the existence of two Higgs doublets $H_u$ and $H_d$ in $\mathbf {5}$ and ${\bar{\mathbf{5}}}$ representations that break electroweak symmetry and give masses to the charge $+$2/3 and charge −1/3 and −1 matter fields, respectively. It is well known that this assumption gives a (reasonably) successful relation between the masses of the *b* quark and the $\tau$ lepton [85–87], but not for the lighter charge −1/3 quarks and charged leptons. We assume that whatever physics resolves this issue is irrelevant for our analysis, as would be the case, for instance, if corrections to the naive SU(5) mass relations were generated by higher-dimensional superpotential terms [88]. In the absence of any phenomenological constraints, we allow the soft SUSY-breaking contributions to the $H_u$ and $H_d$ masses, $m_{H_u}$ and $m_{H_d}$, to be different from each other, as in the NUHM2, as well as from $m_5$ and $m_{10}$. As in the CMSSM, NUHM1 and NUHM2, we allow the ratio of Higgs vacuum expectation values, $\tan \beta$, to be a free parameter.

In addition to these electroweak Higgs representations, we require one or more Higgs representations to break the SU(5) GUT symmetry. The minimal possibility is a single $\mathbf {24}$ representation $\Sigma$, but we do not commit ourselves to this minimal scenario. It is well known that this scenario has problems with rapid proton decay^2^ and GUT threshold effects on gauge coupling unification. We assume that these issues are resolved by the appearance of additional fields at or around the GUT scale that are otherwise irrelevant for TeV-scale phenomenology. The effective low-energy Higgsino mixing coupling $\mu$ is a combination of an input bilinear $H_u H_d$ coupling and possible trilinear and higher-order couplings to GUT-scale Higgs multiplets such as $H_u \Sigma H_d$. We assume that these combine to yield $\mu = {\mathcal O}(1) \,\, \mathrm {TeV}$ and positive, without entering into the possibility of some dynamical mechanism, and commenting below only briefly on the case $\mu < 0$.

## Implementations of constraints and analysis procedure

Our treatments in this paper of many of the relevant constraints follow very closely the implementations in our previous analyses of other supersymmetric models [10–16]. For the convenience of the reader, we summarise the constraints in Table 1. In the following subsections we review our implementations, highlighting new constraints and instances where we implement constraints differently from our previous work.

### Electroweak and flavour constraints

We treat as Gaussian constraints all electroweak precision observables, all *B*-physics and *K*-physics observables except for $\mathrm{BR}(B_{s, d} \rightarrow \mu ^{+}\mu ^{-})$. The $\chi ^2$ contribution from $\mathrm{BR}(B_{s, d} \rightarrow \mu ^{+}\mu ^{-})$, combined here in the quantity $R_{\mu \mu }$ [13], is calculated using a combination of the CMS [89] and LHCb [90] results described in [91] with the more recent result from ATLAS [92]. We extract the corresponding $\chi ^2$ contribution in Table 1 by applying to the two-dimensional likelihood provided by the combination of these experiments the minimal flavour violation (MFV) assumption that applies in the SU(5) model. We calculate the elements of the CKM matrix using only experimental observables that are not included in our set of flavour constraints.

We have updated our implementations of all the flavour constraints, and now use the current world average value of $m_t$ [93]. These and all other constraints whose implementations have been changed are indicated by arrows and boldface in Table 1.

**Table 1:** List of experimental constraints used in this work, including experimental and (where applicable) theoretical errors: supersymmetric theory uncertainties are indicated separately. Instances where our implementations differ from those in Table 1 in [15] are indicated by arrows and boldface

Observable	Source	Constraint
	Th./Ex.	
$\rightarrow \quad \mathbf {m_t}$ [GeV]	[93]	$\mathbf {173.34\pm 0.76}$
$\Delta \alpha _\mathrm{had}^{(5)}(M_Z)$	[94]	$0.02771 \pm 0.00011$
$M_Z$ [GeV]	[95, 96]	$91.1875\pm 0.0021$
$\Gamma _{Z}$ [GeV]	[97, 98]/[95, 96]	$2.4952\pm 0.0023\pm 0.001_\mathrm{SUSY}$
$\sigma _\mathrm{had}^{0}$ [nb]	[97, 98]/[95, 96]	$41.540\pm 0.037$
$R_l$	[97, 98]/[95, 96]	$20.767\pm 0.025$
$A_\mathrm{FB}(\ell )$	[97, 98]/[95, 96]	$0.01714\pm 0.00095$
$A_{\ell }(P_\tau )$	[97, 98]/[95, 96]	0.1465 ± 0.0032
$R_\mathrm{b}$	[97, 98]/[95, 96]	0.21629 ± 0.00066
$R_\mathrm{c}$	[97, 98]/[95, 96]	0.1721 ± 0.0030
$A_\mathrm{FB}({b})$	[97, 98]/[95, 96]	0.0992 ± 0.0016
$A_\mathrm{FB}({c})$	[97, 98]/[95, 96]	0.0707 ± 0.0035
$$ A_{b}$$	[97, 98]/[95, 96]	0.923 ± 0.020
$$ A_{c}$$	[97, 98]/[95, 96]	0.670 ± 0.027
${A_\mathrm{LR}^{e}}$	[97, 98]/[95, 96]	0.1513 ± 0.0021
$\sin ^2 \theta _\mathrm{w}^{\ell }(Q_\mathrm{fb})$	[97, 98] /[95, 96]	0.2324 ± 0.0012
$M_W$ [GeV]	[97, 98]/[95, 96]	$80.385 \pm 0.015\pm 0.010_\mathrm{SUSY}$
$a_{\mu }^\mathrm{EXP} - a_{\mu }^\mathrm{SM}$	[99–106] /[107, 108]	$(30.2 \pm 8.8 \pm 2.0_\mathrm{SUSY})\times 10^{-10}$
$\rightarrow \quad \mathbf {M_h}$ [GeV]	[109–115]/[116]	$\mathbf {125.09} \pm \mathbf{0.24} \pm \mathbf{1.5}_\mathrm{SUSY}$
$\rightarrow \quad \mathbf{BR}_{b \rightarrow s \gamma }^\mathrm{EXP/SM}$	[117]/[118]	$\mathbf {1.021} \pm \mathbf {0.066}_\mathrm{EXP} \pm \mathbf {0.070}_\mathrm{TH,SM} \pm \mathbf {0.050}_\mathrm{TH,SUSY}$
$\rightarrow \quad {\mathbf {R}}_{\mu \mu }$	[119]/[91, 92]	**2D likelihood**, **MFV**
$\rightarrow \quad$ **BR** $_{B \rightarrow \tau \nu }^\mathrm{EXP/SM}$	[118, 120]	$\mathbf {1.02} \pm \mathbf{0.19}_\mathrm{EXP} \pm \mathbf{0.13}_\mathrm{SM}$
$\rightarrow \quad \mathbf {BR}_{B \rightarrow X_s \ell \ell }^\mathrm{EXP/SM}$	[121]/[118]	$\mathbf {0.99} \pm \mathbf{0.29}_\mathrm{EXP} \pm \mathbf{0.06}_\mathrm{SM}$
$\rightarrow \quad \mathbf {BR}_{K \rightarrow \mu \nu }^\mathrm{EXP/SM}$	[122–124]/[94]	$\mathbf {0.9998} \pm \mathbf{0.0017}_\mathrm{EXP} \pm \mathbf{0.0090}_\mathrm{TH}$
$\rightarrow \quad \mathbf {BR}_{K \rightarrow \pi \nu {\bar{\nu }}}^\mathrm{EXP/SM}$	[125]/[126]	$\mathbf {2.2} \pm \mathbf{1.39}_\mathrm{EXP} \pm \mathbf{0.20}_\mathrm{TH}$
$\rightarrow \quad \mathbf {\Delta M}_{B_s}^\mathrm{EXP/SM}$	[122, 123, 127]/[118]	$\mathbf {1.016} \pm \mathbf{0.074}_\mathrm{SM}$
$\rightarrow \quad {\frac{\mathbf {\Delta M}_{B_s}^\mathrm{EXP/SM}}{\Delta \mathbf{M}_{B_{d}}}^\mathrm{EXP/SM}}$	[122, 123, 127]/[118]	$\mathbf {0.84} \pm \mathbf{0.12}_\mathrm{SM}$
$\rightarrow \quad \mathbf {\Delta \epsilon }_K^\mathrm{EXP/SM}$	[122, 123, 127]/[94]	$\mathbf {1.14} \pm \mathbf{0.10}_\mathrm{EXP+TH}$
$\rightarrow \quad \mathbf {\Omega _{CDM} h^2}$	[128, 129]/[74]	$\mathbf {0.1186} \pm \mathbf{0.0020}_\mathrm{EXP} \pm \mathbf {0.0024}_\mathrm{TH}$
$\rightarrow \mathbf {\sigma ^\mathrm{SI}_p}$	[83, 84]	$(\mathbf{m}_{{\tilde{\chi }}_\mathbf{1}^\mathbf{0}}, {\sigma ^\mathrm{SI}_\mathbf{p}})$ **plane**
$\rightarrow \quad$ **Heavy stable charged particles**	[130]	**Fast simulation based on** [130, 131]
$\rightarrow \quad {\tilde{\mathbf{q}}} \rightarrow \mathbf{q} \tilde{\chi }^\mathbf{0}_\mathbf{1} , {\tilde{\mathbf{g}}} \rightarrow f {\bar{f}} \tilde{\chi }^\mathbf{0}_\mathbf{1}$	[9]	$\mathbf {\sigma } \cdot \mathrm{BR}$ **limits in the** $({\mathbf{m}_{\tilde{\mathbf{q}}}, \mathbf{m}_{{\tilde{\chi }}_\mathbf{1}^\mathbf{0}})}, {(\mathbf{m}_{\tilde{\mathbf{g}}}, \mathbf{m}_{{\tilde{\chi }}_\mathbf{1}^\mathbf{0}})}$ **planes**
$\rightarrow \mathbf {H/A \rightarrow \tau ^+ \tau ^-}$	[132–134]	**2D likelihood**, ${\sigma } \cdot \mathrm{BR}$ **limit**

### Higgs constraints

We use the combination of ATLAS and CMS measurements of the mass of the Higgs boson: $M_h= 125.09 \pm 0.24 \,\, \mathrm {GeV}$ [116]. We employ the FeynHiggs 2.11.2 code [109–115] to evaluate the constraint this imposes on the parameter space, assuming a one-$\sigma$ theoretical uncertainty of $1.5 \,\, \mathrm {GeV}$.^3^

The $\chi ^2$ contributions of 85 Higgs search channels from the LHC and the Tevatron are evaluated using HiggsSignals, see [136, 137], where a complete list of references can be found. The $\chi ^2$ contributions from the limits from searches for the heavy neutral MSSM Higgs bosons in the $H/A \rightarrow \tau ^+\tau ^-$ channels are evaluated using the code HiggsBounds  [133, 138–140], which incorporates the results of CMS searches [132, 133] with ${\sim } 25~\mathrm{fb}^{-1}$ of 8 TeV data. The contributions from the two possible production modes, $gg \rightarrow H/A$ and $b \bar{b} \rightarrow H/A$, are combined in a consistent manner, depending on the MSSM parameters. The results from HiggsBounds have been compared with the published CMS analysis, and are in very good agreement [133]. The corresponding $\chi ^2$ contribution is labelled “2D likelihood” in Table 1. For the corresponding constraint with 13 fb$^{-1}$ of 13 TeV data, we implement an approximate treatment of the $\chi ^2$ contribution using the preliminary result of ATLAS [134], as we describe in more detail below. Limits from other Higgs boson searches are not relevant for the investigation in this paper and are therefore not included.

### LHC  constraints at 13 TeV

ATLAS and CMS have recently announced preliminary results from  searches with ${\sim } 13$/fb of data at 13 TeV, using simplified models for gluino and squark pair production [5, 9]. These searches assume $m_{\tilde{g}}\ll m_{\tilde{q}}$ and $m_{\tilde{q}}\ll m_{\tilde{g}}$, respectively, and 100% BRs for the decays ${\tilde{g}}\rightarrow f {\bar{f}} \tilde{\chi }^{0}_{1}$ ($f = q, b, t$) and $\tilde{q}\rightarrow q \tilde{\chi }^{0}_{1}$, respectively, which maximize the possible corresponding  signatures. Neither of these assumptions is valid in the SUSY SU(5) GUT model: as we will see in more detail later, the $m_{\tilde{g}}$ and $m_{\tilde{q}}$ masses are quite similar in much of the favoured region of parameter space,^4^ and in general other decay modes dilute the  signature, although larger-multiplicity final states may compensate through an increase in transverse energy $H_T$ [141]. These other decay modes populate other search channels including leptons, which we do not consider in this paper as they were of limited importance in our previous analyses of the CMSSM, NUHM1 and NUHM2, having impact only for relatively large squark masses and small $m_{1/2}$.

**Fig. 1:**
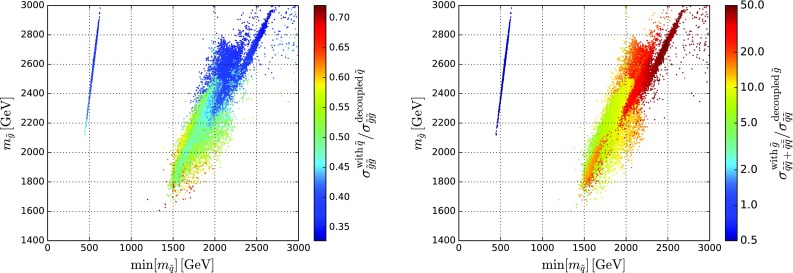
*Left panel* the ratio of the ${\tilde{g}}{\tilde{g}}$ cross section that we find in the range of $m_{\tilde{q}}$ and $m_{\tilde{g}}$ favoured in our analysis before implementing the LHC 13-TeV  constraint, relative to the cross section found in the simplified model with $m_{\tilde{g}}\ll m_{\tilde{q}}$. *Right panel* the corresponding ratio of the $\tilde{q}\tilde{q}+ \tilde{q}\bar{\tilde{q}}$ cross section, relative to the cross section for $\tilde{q}\bar{\tilde{q}}$ found in the simplified model with $m_{\tilde{q}}\ll m_{\tilde{g}}$

Figure 1 displays the ratios of the ${\tilde{g}}{\tilde{g}}$ cross section (left panel) and the $\tilde{q}\tilde{q}+ \tilde{q}\bar{\tilde{q}}$ cross section (right panel) that we find in ranges of $m_{\tilde{q}}$ and $m_{\tilde{g}}$ that are representative of those favoured in our analysis before implementing the LHC 13-TeV  constraint, relative to the cross sections found in the simplified models with $m_{\tilde{g}}\ll m_{\tilde{q}}$ and $m_{\tilde{q}}\ll m_{\tilde{g}}$, respectively. We have used NLL-fast-3.1 [142] to obtain the cross section at NLO $+$ NLL level. In both plots a large area at higher squark masses is visible, as well as a thin strip at ${\sim } 500 \,\, \mathrm {GeV}$. The latter corresponds to lighter ${\tilde{u}_R}$ and ${\tilde{c}_R}$ discussed below. We see that the ${\tilde{g}}{\tilde{g}}$ cross section (left panel) is generally *smaller* than in the corresponding simplified model by a factor ${>} 2$ due to the destructive interference between the *s*-channel gluon exchange diagram and the *t*-channel squark exchange diagram in $q \bar{q} \rightarrow {\tilde{g}}{\tilde{g}}$, thus weakening the LHC constraints as discussed below. On the other hand, the $\tilde{q}\tilde{q}+ \tilde{q}\bar{\tilde{q}}$ cross section (right panel) is generally *a factor* $\gtrsim 10$ *larger* than in the simplified model, except in the ${\tilde{u}_R}/{\tilde{c}_R} - \tilde{\chi }^{0}_{1}$ coannihilation strip at small $m_{\tilde{u}_R}, m_{\tilde{c}_R}, m_{\tilde{\chi }^{0}_{1}} \sim 500 \,\, \mathrm {GeV}$ and $m_{1/2} \sim 2500 \,\, \mathrm {GeV}$, to which we return later. The enhancement of the squark cross section is due to the fact that in the squark-neutralino simplified model there is no production mode with total baryon number $B = 2/3$, $qq \rightarrow \tilde{q} \tilde{q}$, because gluinos are assumed to be absent. On the other hand, in our model $m_{\tilde{g}}\sim \min (m_{\tilde{q}})$, and $qq \rightarrow \tilde{q} \tilde{q}$ (with *t*-channel ${\tilde{g}}$ exchange) becomes the dominant squark production mode in the large $m_{\tilde{q}}$ region, due to the valence quark-parton dominance in the proton in the large *x* regime.

**Fig. 2:**
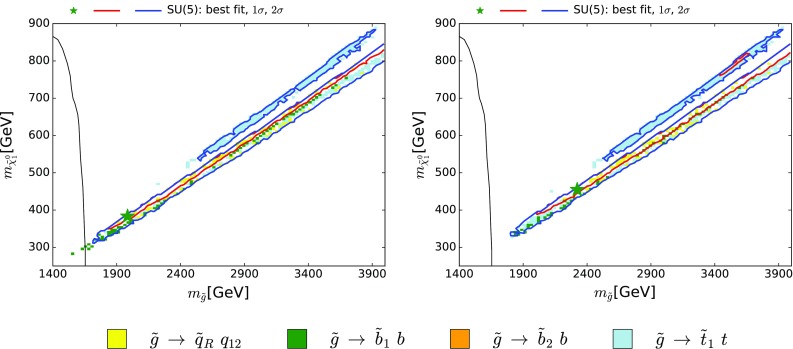
The *solid lines* show the CMS 95% CL exclusion in the $(m_{\tilde{g}}, m_{\tilde{\chi }^{0}_{1}})$ plane [9], assuming a simplified model with heavy squarks and 100% BR for ${\tilde{g}}\rightarrow q {\bar{q}} \tilde{\chi }^{0}_{1}$. The *left* (*right*) *panel* shows the best-fit point (*green star*), 68 and 95% CL contours (*red* and *blue lines*, respectively) for $(m_{\tilde{g}}, m_{\tilde{\chi }^{0}_{1}})$ obtained without (with) the CMS 13-TeV constraint. The dominant (>50%) ${\tilde{g}}$ decays into first- and second-generation quarks and squarks $\tilde{q}_{L,R}$ and third-generation quarks and squarks ${\tilde{t}/\tilde{b}}_{1,2}$ found in the SUSY SU(5) model are *colour-coded* as indicated

Figure 2 displays the CMS 95% confidence limits in the $(m_{\tilde{g}}, m_{\tilde{\chi }^{0}_{1}})$ plane from a hadronic jets plus  search [9] within a simplified model assuming that the decay mode ${\tilde{g}}\rightarrow q {\bar{q}} \tilde{\chi }^{0}_{1}$ occurs with 100% BR (solid black lines). These limits are compared with the best-fit points (green stars) and the regions in the fits that are preferred at $\Delta \chi ^2 = 2.30$ and $\Delta \chi ^2 = 5.99$ (red and blue contours, respectively). Here and in the following analogous parameter planes, we use the $\Delta \chi ^2 = 2.30$ and $\Delta \chi ^2 = 5.99$ contours as proxies for the boundaries of the 68 and 95% CL regions in the fit.

In addition, within the 95% CL region in Fig. 2 we have indicated the dominant (${>} 50$%) ${\tilde{g}}$ decays found in our analysis. We note that many model points do not have any decay mode with BR ${>} 50$% within the 95% CL region and that, for those that do, the dominant decays are two-body ${\tilde{g}}\rightarrow \tilde{q}{\bar{q}}$ modes that were not considered in [9]. Because of this and the fact that the ${\tilde{g}}{\tilde{g}}$ cross section is always smaller than in the gluino simplified model by a factor $> 2$ (see the left panel of Fig. 1), the LHC 13-TeV  constraint from the gluino simplified model has only negligible impact. Our LHC 13-TeV  constraint on the gluino mass actually comes indirectly from the squark mass constraint estimated using the squark simplified model discussed below, since the squark and gluino masses are related via renormalization group evolution in the SU(5) model. The left panel in Fig. 2 was obtained before implementing the LHC 13-TeV  95% confidence limit on gluino and squark pair production, while in the right panel this constraint is included. We note that the simplified-model exclusion in this analysis extended to $m_{\tilde{g}}\lesssim 1650 \,\, \mathrm {GeV}$, below the gluino mass at the pre-LHC 13 TeV best-fit point, and barely reaching the 68% CL contour (solid red line).

**Fig. 3:**
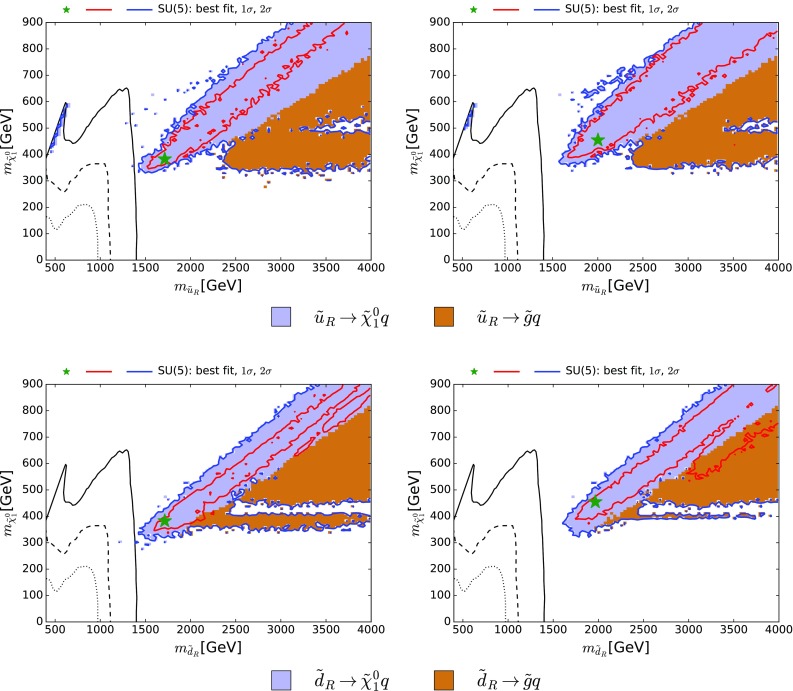
The *black lines* show the CMS 95% CL exclusion in the $(m_{\tilde{q}}, m_{\tilde{\chi }^{0}_{1}})$ plane [9], assuming a simplified model with heavy gluinos and 100% BR for $\tilde{q}\rightarrow q \tilde{\chi }^{0}_{1}$: the *solid lines* assume that all the squarks of the first two generations are degenerate, the *dashed lines* assume two degenerate squarks, and the *dotted lines* assume just one squark. *All panels* show the best-fit point (*green star*), 68 and 95% CL contours (*red* and *blue lines*, respectively) for $m_{\tilde{\chi }^{0}_{1}}$ and the masses of the first- and second-generation right-handed up-type squarks $\tilde{u}_{R}$ (*upper panels*) and the down-type squarks $\tilde{d}_{R}$ (*lower panels*). In both cases, the *left panels* were obtained without the CMS 13-TeV constraint, and the *right panels* include it. The dominant (>50%) $\tilde{q}$ decays found in the SUSY SU(5) model are *colour-coded* as indicated

Figure 3 contains an analogous set of planes for CMS  searches for squarks, where the CMS limit assuming a simplified model with heavy gluino and 100% BRs for $\tilde{q}\rightarrow q \tilde{\chi }^{0}_{1}$ is displayed (black lines): the solid lines assume that all the squarks of the first two generations are degenerate, the dashed lines assume two degenerate squarks, and the dotted lines assume just one squark. The planes in the upper panels display $m_{\tilde{\chi }^{0}_{1}}$ and the masses of the first- and second-generation right-handed up-type squarks (here commonly denoted $\tilde{u}_{R}$), while the planes in the lower panels are for the down-type squarks (here commonly denoted $\tilde{d}_{R}$). The main decay modes of the $\tilde{u}_R$ (upper) and the $\tilde{d}_R$ (lower) are indicated over much of the preferred parameter space, and we note that the dominant (${>} 50$%) decay modes of both right-handed up- and down-type squarks are indeed into the corresponding quark flavour $+$ $\tilde{\chi }^{0}_{1}$ for nearly the whole 68% CL regions, as assumed in the squark simplified-model search. This is, however, not the case for the left-handed up- and down-type squarks (not shown), whose dominant decays are into $\tilde{\chi }^{\pm }_{1}$ and electroweak doublet partner quark flavours. Furthermore, within the displayed 95% CL regions there are also large areas where decays into gluinos, not considered in the simplified model, are dominant.

Because the ${\tilde{q}_R} \rightarrow q \, \tilde{\chi }^{0}_{1}$ decays are important, and also because the $\tilde{q}\tilde{q}+ \tilde{q}\bar{\tilde{q}}$ cross section in our sample is much larger than that found at large $m_{\tilde{q}}$ for $\tilde{q}\bar{\tilde{q}}$ in the simplified model with $m_{\tilde{q}}\ll m_{\tilde{g}}$, as seen in the right panel of Fig. 1, we have implemented a recast of this search in our global analysis,^5^ and the comparison between the left panels (without this contribution) and the right panels (with this contribution) in Fig. 3 shows the importance of this constraint.

Our implementation of the LHC 13-TeV  constraint is based on [9]. In this analysis, the CMS Collaboration provides a map of the 95% CL cross-section upper limit as a function of $m_{\tilde{q}}$ and $m_{{\tilde{\chi }}_1^0}$ assuming $pp \rightarrow \tilde{q} \bar{\tilde{q}}$ and 100% BR for $\tilde{q} \rightarrow q {\tilde{\chi }}_1^0$. This is indeed the dominant production and decay mode in most parts of the 68% CL regions of the considered model, as can be seen in Figs. 1 and 3. For each point we compare our calculation of $(\sigma _{\tilde{q}{\bar{\tilde{q}}}} + \sigma _{\tilde{q}\tilde{q}})\;\mathrm{BR}^2_{\tilde{q} \rightarrow q {\tilde{\chi }}_1^0}$ with the CMS 95% CL upper limit on the cross section: $\sigma _\mathrm{UL}(m_{\tilde{q}, {\tilde{\chi }}_1^0})$. We model the $\chi ^2$ penalty as2so that the CMS 95% CL upper limit corresponds to  and $\chi ^2$ scales as the square of the number of signal events, $N_\mathrm{sig}$, which gives the right scaling. We have checked that our implementation (2) reproduces the ${\pm } 1 \,\sigma$ band in the two-dimensional exclusion limit provided by CMS [9], with a discrepancy that is much smaller than the width of the ${\pm } 1 \,\sigma$ band.

The aforementioned CMS analysis [9] also looks at three simplified gluino models assuming 100% BR for $\tilde{g} \rightarrow f \bar{f} {\tilde{\chi }}_1^0$ with $f = q, b, t$, respectively, and provides corresponding cross-section upper limit maps as a function of $m_{\tilde{g}}$ and $m_{{\tilde{\chi }}_1^0}$. We implement these constraints by defining  by analogy with Eq. (2).

We also consider the $pp \rightarrow \tilde{q} \tilde{g}$ process, treating it as follows. This process is only relevant when $m_{\tilde{q}}\sim m_{\tilde{g}}$. In this regime, if $m_{\tilde{q}}> m_{\tilde{g}}$ ($m_{\tilde{g}}> m_{\tilde{q}}$), $\tilde{q}$ ($\tilde{g}$) tends to decay into $\tilde{g}$ ($\tilde{q}$), radiating soft jets. If these soft jets are ignored, we are left with the ${\tilde{g}}{\tilde{g}}$ ($\tilde{q}\tilde{q}$) system. In this approximation, the impact of $pp \rightarrow \tilde{q} \tilde{g}$ can therefore be estimated by adding an extra contribution $\sigma _{\tilde{q} \tilde{g}} BR_{\tilde{q} \rightarrow q \tilde{g}}$ ($\sigma _{\tilde{q} \tilde{g}} BR_{\tilde{g} \rightarrow q \tilde{q}}$) to $\sigma _{{\tilde{g}}{\tilde{g}}}$ ($\sigma _{\tilde{q}\tilde{q}} + \sigma _{\tilde{q}\bar{\tilde{q}}}$). In general, SUSY searches are designed to look for high $p_T$ objects, and one loses a small amount of sensitivity by ignoring soft jets. We therefore believe that our implementation of the $pp \rightarrow \tilde{q} \tilde{g}$ process is conservative.

Finally, we estimate the total $\chi ^2$ penalty from the LHC 13-TeV  constraint to be .^6^

### Constraints on long-lived charged particles

We also include in our analysis LHC constraints from searches for heavy long-lived charged particles (HLCP) that are, in general, relevant to coannihilation regions where the mass difference between the lightest SUSY particle (LSP) and the next-to-lightest SUSY particle (NLSP) may be small and the NLSP may therefore be long-lived. As we discuss below, important roles are played in our analysis by ${\tilde{\tau }}_1$, $\tilde{\chi }^{\pm }_{1}$ and $\tilde{u}_R/\tilde{c}_R$ coannihilation, but only in the ${\tilde{\tau }}_1$ case is the NLSP - LSP mass difference small enough to offer the possibility of a long-lived charged particle. We implement in our global analysis the preliminary CMS 13-TeV result [130] using tracking and time-of-flight measurements, based on the recipe and the efficiency map as a function of the pseudo-rapidity and velocity of the HLCP given in [131]. We use Pythia 8 [143] and Atom [144–148] to generate and analyse the events, and assume that the efficiencies for detecting slow-moving ${\tilde{\tau }}_1$s are similar at 8 and 13 TeV.^7^ The efficiency contains a lifetime-dependent factor $\propto \mathrm{exp}(- d m/ p \tau )$, where *d* is a distance $d \simeq 10$ m that depends on the pseudorapidity, and *m*, *p* and $\tau$ are the mass, momentum and lifetime of the long-lived particle. This factor drops rapidly for particles with lifetimes ${\lesssim } 10$ ps, corresponding to $m_{{\tilde{\tau }}_1} - m_{\tilde{\chi }^{0}_{1}} \gtrsim 1.6 \,\, \mathrm {GeV}$.

### Constraints on heavy neutral Higgs bosons from Run II

Concerning the production of heavy neutral Higgs bosons, in addition to the 8 TeV constraints on $H/A \rightarrow \tau ^+ \tau ^-$ provided by HiggsBounds, we also take into account the preliminary exclusion limits obtained by ATLAS from searches for generic spin-0 bosons $\phi$ in the $\tau \tau$ final state with an integrated luminosity of 13.3 fb$^{-1}$ at 13 TeV that were presented at the ICHEP 2016 conference and described in [134] (see also the CMS results in [135]). Upper bounds on $\sigma \times \mathrm{BR}({\phi } \rightarrow \tau \tau )$ are reported for each ${M_\phi }$ separately for the gluon fusion production channel and for production in association with a $b {\bar{b}}$ pair assuming there is no contamination between the modes, assuming a single resonance. We compute the cross sections and the BRs in the MSSM using FeynHiggs, adding the contributions for $\phi = H$ and $\phi = A$, using the average of the two masses, which are degenerate within the experimental resolution. This result is compared with the upper limit from the corresponding channel neglecting contamination. This approach leads to a conservative limit since we underestimate the signal yield in each channel by neglecting the contamination (the events from the other production mode). As in Eq. (2), the $\chi ^2$ penalties are modelled as

3 $$\begin{aligned} \chi ^2({Y_i}) = 4 \cdot \left( \frac{ \sigma _{X_i} \cdot \mathrm{BR}_{\tau ^+ \tau ^-} }{ \sigma _{Y_i}^\mathrm{UL}(M_A) } \right) ^2, \end{aligned}$$

where $X_i = (gg \rightarrow {H/A}$, $pp \rightarrow b \bar{b} {H/A})$ is the production mode, $Y_i = (ggF, bb{\phi })$ is the corresponding search channel and $\sigma ^\mathrm{UL}(M_A)$ is the 95% CL upper limit evaluated at $M_A{({\approx } M_H)}$ by ATLAS [134]. Finally we take the stronger $\chi ^2$ rather than combining them, in order to be on the conservative side^8^: $\chi ^2({H/A \rightarrow \tau ^+ \tau ^-}) = \max ( \chi ^2({ggF}), \chi ^2({bb{\phi }}) )$.

**Table 2:** Ranges of the SUSY SU(5) GUT parameters sampled, together with the numbers of segments into which each range was divided, and the corresponding total number of sample boxes. The mass parameters are expressed in TeV units

Parameter	Range	Number of segments
$m_{1/2}$	(0, 4)	2
$m_{5}$	(−2.6, 8)	2
$m_{10}$	(−1.3, 4)	3
$m_{H_u}$	(−7, 7)	3
$m_{H_d}$	(−7, 7)	3
$A_0$	(−8, 8)	1
$\tan {\beta }$	(2, 68)	1
Total number of boxes		108

### Other constraints

The most important other constraint update is that on spin-independent DM scattering. We incorporate in our global fit the recent result published by the PandaX-II experiment [83], which we combine with the new result from the LUX Collaboration [84], as discussed in more detail in Sect. 8.

For the electroweak observables we use FeynWZ [97, 98], and for the flavour constraints we use SuFla [122, 123]. For the Higgs observables, we use FeynHiggs 2.11.2  [109–115] (including the updates discussed in Sect. 3.2), HiggsBounds 4.3.1 [133, 138–140] and HiggsSignals 1.4.0 [136, 137]. We calculate the sparticle spectrum using SoftSusy 3.3.10 [151] and sparticle decays using SDECAY 1.3b [152] and StauDecay 0.1 [82]. The DM density and scattering rate are calculated using micrOMEGAs 3.2 [128] and SSARD [129], respectively. Finally, we use SLHALib 2.2 [153, 154] to interface the different codes.

### Sampling procedure

As discussed in the previous Section, the SUSY SU(5) GUT model we study has seven parameters: $m_{1/2}$, $m_5$, $m_{10}$, $m_{H_u}$, $m_{H_d}$, $A_0$ and $\tan {\beta }$. The ranges of these parameters that we scan in our analysis are listed in Table 2. The quoted negative values actually correspond to negative values of $m^2_5, m^2_{10}, m^2_{H_u}$ and $m^2_{H_d}$: for convenience, we use the notation $\mathrm{sign}(m^2) \times \sqrt{|m^2|} \rightarrow m$. The negative values of $m_5$ and $m_{10}$ that are included in the scans may be compatible with early-Universe cosmology [155], and yield acceptable tachyon-free spectra. In the portions of the scans with negative values of $m_{H_u}$ and $m_{H_d}$, although the effect of the top quark Yukawa coupling in the renormalization group equations is important, it may not be the mechanism responsible for generating electroweak symmetry breaking, since $m_{H_u}$ and $m_{H_d}$ are negative already at the input scale.

We sample this parameter space using MultiNest v2.18 [156–158], dividing the seven-dimensional parameter space into 108 boxes, as also described in Table 2. This has two advantages: it enables us to run MasterCode on many nodes in parallel, and it enables us to probe more efficiently for local features in the likelihood function. For each box, we choose a prior such that 80% of the sample has a flat distribution within the nominal range, while 20% of the sample is in normally distributed tails outside the box. Our resultant total sample overlaps smoothly between boxes, avoiding any spurious features at the box boundaries. The total number of points in our sample is ${\sim } 125 \times 10^6$, of which ${\sim } 8 \times 10^6$ have $\Delta \chi ^2 < 10$.

## Dark matter mechanisms

The relic density of the LSP, assumed here to be the lightest neutralino, $\tilde{\chi }^{0}_{1}$, which is stable in supersymmetric SU(5) because of *R*-parity, may be brought into the narrow range allowed by the Planck satellite and other measurements [74] via a combination of different mechanisms. It was emphasized previously [16] in studies of the CMSSM, NUHM1 and NUHM2 that simple annihilations of pairs of LSPs into conventional particles would not have been sufficient to bring the relic $\tilde{\chi }^{0}_{1}$ density down into the Planck range for values of $m_{\tilde{\chi }^{0}_{1}}$ compatible with the LHC search limits and other constraints on these models. Instead, there has to be some extra mechanism for suppressing the LSP density. Examples include enhanced, rapid annihilation through direct-channel resonances such as *Z*, *h*, *H* / *A*. Another possibility is coannihilation with some other, almost-degenerate sparticle species  [18, 159–162]: candidates for the coannihilating species identified in previous studies include the ${\tilde{\tau }}_1, {\tilde{\mu }}, {\tilde{e}}, {{\tilde{\nu }}}, {\tilde{t}_1}$ and $\tilde{\chi }^{\pm }_{1}$.

**Fig. 4:**
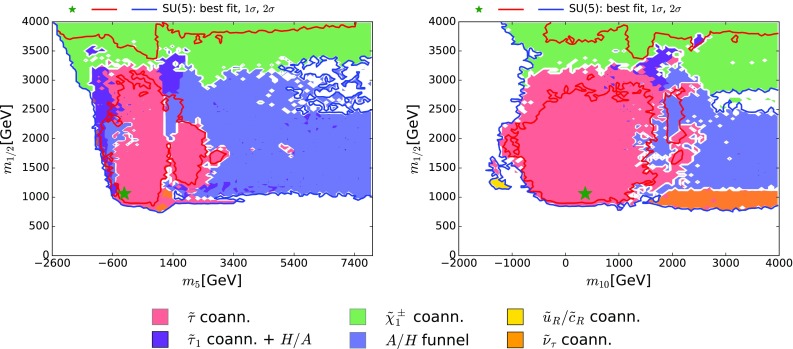
The $(m_5, m_{1/2})$ plane (*left panel*) and the $(m_{10}, m_{1/2})$ plane (*right panel*) in the SUSY SU(5) GUT model. The best-fit point is shown as a *green star*, the *red contour* surrounds the 68% CL region, and the *blue contour* surrounds the 95% CL region. The *coloured shadings* represent the dominant DM mechanisms, as indicated in the *lower panel* and described in the text

We introduced in [16] measures on the sparticle mass parameters that quantify the mass degeneracies relevant to the above-mentioned coannihilation and rapid annihilation processes, of which the following are relevant to our analysis of the SUSY SU(5) GUT model^9^:

4 $$\begin{aligned} {{\tilde{\tau }}_1} \mathrm{~coann.~(pink):} \left( \frac{m_{{\tilde{\tau }}_1}}{m_{\tilde{\chi }^{0}_{1}}} - 1 \right)&< 0.15, \nonumber \\ \tilde{\chi }^{\pm }_{1} \mathrm{~coann.~(green):} \left( \frac{m_{\tilde{\chi }^{\pm }_{1}}}{m_{\tilde{\chi }^{0}_{1}}} - 1 \right)&< 0.1, \nonumber \\ A/H \mathrm{~funnel~(pale~blue):} \left| \frac{M_A}{m_{\tilde{\chi }^{0}_{1}}} - 2 \right|&< 0.4. \end{aligned}$$

We also indicate above the colour codes used in subsequent figures to identify regions where each of these degeneracy conditions applies. We have verified in a previous study [16] that CMSSM, NUHM1 and NUHM2 points that satisfy the DM density constraint fulfill one or more of the mass-degeneracy conditions, and that they identify correctly the mechanisms that yield the largest fractions of final states, which are usually ${\gtrsim } 50$% [14, 169].

In much of the region satisfying the ${\tilde{\tau }}_1$ degeneracy criterion above, the ${{\tilde{\nu }}_\tau }$ has a similar mass, and can contribute to coannihilation [71]. We highlight the parts of the sample where sneutrino coannihilation is important by introducing a shading for regions where the ${{\tilde{\nu }}_\tau }$ is the next-to-lightest sparticle (NLSP), and it obeys the degeneracy condition

5 $$\begin{aligned} {{\tilde{\nu }}_\tau }^\mathrm{NLSP} \mathrm{~coann.~(orange):} \left( \frac{m_{{\tilde{\nu }}_\tau }}{m_{\tilde{\chi }^{0}_{1}}} - 1 \right) < 0.1. \end{aligned}$$

We discuss later the importance of this supplementary DM mechanism.

As we discuss in this paper, a novel possibility in the SU(5) SUSY GUT is coannihilation with right-handed up-type squarks, ${\tilde{u}_R}$ and ${\tilde{c}_R}$, which may be much lighter than the other squarks in this model, as a consequence of the freedom to have $m_5 \ne m_{10}$. We quantify the relevant mass degeneracy criterion by

6 $$\begin{aligned} {\tilde{u}_R/\tilde{c}_R} \mathrm{~coann.~(yellow):} \; \left( \frac{m_{\tilde{u}_R/\tilde{c}_R}}{m_{\tilde{\chi }^{0}_{1}}} - 1 \right) < 0.2. \end{aligned}$$

As we shall see in the subsequent figures, this novel degeneracy condition can play an important role when $m_5 \gg m_{10}$. The existence of this new coannihilation region was verified using SSARD [129], an independent code for calculating the supersymmetric spectrum and relic density.

We also distinguish in this analysis ‘hybrid’ regions where the ${\tilde{\tau }}_1$ coannihilation and *H* / *A* funnel mechanisms may be relevant simultaneously:

7 $$\begin{aligned} {\tilde{\tau }}_1~\mathrm{coann.} + H/A~\mathrm{funnel:} \quad \mathrm{(purple)}, \end{aligned}$$

also with the indicated colour code.

## Results

### Parameter planes

We display in Fig. 4 features of the global $\chi ^2$ function for the SUSY SU(5) GUT model in the $(m_5, m_{1/2})$ plane (left panel) and the $(m_{10}, m_{1/2})$ plane (right panel), profiled over the other model parameters.^10^ Here and in subsequent parameter planes, the best-fit point is shown as a green star, the 68% CL regions are surrounded by red contours, and the 95% CL regions are surrounded by blue contours (as mentioned above, we use the $\Delta \chi ^2 = 2.30$ and $\Delta \chi ^2 = 5.99$ contours as proxies for the boundaries of the 68 and 95% CL regions in the fit). The regions inside the 95% CL contours are shaded according to the dominant DM mechanisms discussed in the previous section, see the criteria (4, 6, 7). In the (relatively limited) unshaded regions there is no single dominant DM mechanism.

As we see in Fig. 4, the best-fit point is at relatively small values of $m_5, m_{10}$ and $m_{1/2}$, close to the lower limit on $m_{1/2}$, whereas the 68% CL region extends to much larger values of $m_5, m_{10}$ and $m_{1/2}$. The values of the model parameters at the best-fit point are listed in Table 3.^11^ The upper row of numbers are the results from the current fit including the latest LHC 13-TeV and PandaX-II/LUX constraints, and the numbers in parentheses in the bottom row were obtained using instead the previous LHC 8-TeV and XENON100 constraints, but the same implementations of the other constraints. The most significant effect of the new LHC data has been to increase the best-fit value of $m_{1/2}$ by ${\sim } 160 \,\, \mathrm {GeV}$: the changes in the other fit parameters are not significant, in view of the uncertainties. As we discuss in more detail later, the favoured fit regions are driven by the $(g-2)_\mu$ constraint towards the boundary of the region excluded by the  constraint. Away from this boundary, the global $\chi ^2$ function is quite flat.

The best-fit point and much of the 68% CL region lie within the pink shaded region where ${\tilde{\tau }}_1- \tilde{\chi }^{0}_{1}$ coannihilation is the dominant DM mechanism. At larger values of $m_5$ and $m_{10}$ we encounter a blue shaded region where rapid annihilation via direct-channel *H* / *A* poles is dominant. We also see darker shaded hybrid regions where ${\tilde{\tau }}_1$ and *H* / *A* annihilation are important simultaneously. At larger values of $m_{1/2} \gtrsim 3000 \,\, \mathrm {GeV}$, in the green shaded regions, the dominant DM mechanism is $\tilde{\chi }^{\pm }_{1} - \tilde{\chi }^{0}_{1}$ coannihilation. There is also a band in the $(m_{10}, m_{1/2})$ plane with $m_{10} \gtrsim 1500 \,\, \mathrm {GeV}$ and $m_{1/2} \sim 1000 \,\, \mathrm {GeV}$, allowed at the 95% CL, where ${{\tilde{\nu }}_\tau }^\mathrm{NLSP}$ coannihilation is important.

**Table 3:** Parameters of the best-fit point in the SUSY SU(5) GUT model, with mass parameters given in GeV units. The numbers in parentheses in the bottom row are for a fit that does not include the LHC 13-GeV constraints and the recent PandaX-II and LUX constraints on DM scattering. Note that we use the same convention for the sign of $A_0$ as in [10–16], which is opposite to the convention used in, e.g., SoftSUSY, and that we use the notation $\mathrm{sign}(m^2) \times \sqrt{|m^2|} \rightarrow m$ for $m_5, m_{10}, m_{H_u}$ and $m_{H_d}$

$m_{1/2}$	$m_5$	$m_{10}$	$m_{H_u}$	$m_{H_d}$	$A_0$	$\tan {\beta }$
1050	−220	380	−5210	−4870	−5680	12
(890)	(−80)	(310)	(−4080)	(−4420)	(5020)	(11)

We also note the appearance within the 95% CL region at $m_{1/2} \sim 1000 \,\, \mathrm {GeV}$, and $m_{10} \sim - 1000 \,\, \mathrm {GeV}$ of the novel ${\tilde{u}_R}/{\tilde{c}_R} - \tilde{\chi }^{0}_{1}$ coannihilation region (shaded yellow). To understand the origin of this novelty, consider the one-loop renormalization-group equations for the states in the $\mathbf {10}$ representations of SU(5), namely $(q_L, u^c_L, e^c_L)_i$, above the highest MSSM particle mass (all masses are understood to be scalar fermion masses, and we suppress subscripts $_L$):

8 $$\begin{aligned} 16 \pi ^2 \frac{\partial m^2_{q_i}}{\partial t}= & {} \delta _{i3} (X_t + X_b) - \frac{32}{3} g_3^2 |M_3|^2 \nonumber \\&- 6 g_2^2 |M_2|^2 - {\frac{2}{15}}g_1^2 |M_1|^2 + \frac{1}{5} g_1^2 S, \end{aligned}$$

9 $$\begin{aligned} 16 \pi ^2 \frac{\partial m^2_{u_i^c}}{\partial t}= & {} {2} \delta _{i3} X_t - \frac{32}{3} g_3^2 |M_3|^2 \nonumber \\&- \frac{32}{15} g_1^2 |M_1|^2 - \frac{4}{5} g_1^2 S, \end{aligned}$$

10 $$\begin{aligned} 16 \pi ^2 \frac{\partial m^2_{e_i^c}}{\partial t}= & {} {2} \delta _{i3} X_\tau - \frac{24}{5} g_1^2 |M_1|^2 + \frac{6}{5} g_1^2 S, \end{aligned}$$

where $t \equiv \ln (Q/Q_0)$ with *Q* the renormalization scale and $Q_0$ some reference scale,

11 $$\begin{aligned} X_t\equiv & {} 2|y_t|^2 (m^2_{H_u} + m^2_{q_3} + m^2_{t^c}) + 2|A_t|^2, \end{aligned}$$

12 $$\begin{aligned} X_b\equiv & {} 2|y_b|^2 (m^2_{H_d} + m^2_{q_3} + m^2_{b^c}) + 2|A_b|^2, \end{aligned}$$

13 $$\begin{aligned} X_\tau\equiv & {} 2|y_\tau |^2 (m^2_{H_d} + m^2_{l_3} + m^2_{\tau ^c}) + 2|A_\tau |^2, \end{aligned}$$

and

14 $$\begin{aligned} S\equiv & {} (m^2_{H_u} - m^2_{H_d}) \nonumber \\&+\, \mathrm{Tr} ({m^2_{q} - m^2_{l}} - 2 m^2_{u^c} + m^2_{d^c} + m^2_{e^c}), \end{aligned}$$

where the trace in *S* sums over the generations. The ${\tilde{u}_R}/{\tilde{c}_R} - \tilde{\chi }^{0}_{1}$ coannihilation mechanism becomes important in a region of the SUSY SU(5) GUT parameter space where $m_5^2$ is very large and positive (${\sim } 27$ TeV$^2$), $m_{10}^2$ is small and negative (${\sim } -1.4$ TeV$^2$), $m_{H_u}^2$ is very large and negative (${\sim } -23$ TeV$^2$), and $m_{H_d}^2$ is very large and positive (${\sim } 50$ TeV$^2$). In this region, therefore, $X_t$ is very large and negative (${\sim } -35$ TeV$^2$), $X_b$ and $X_\tau$ are suppressed because of small Yukawa couplings ($\tan {\beta }$ is not large in this region), and *S* is also very large and negative (${\sim } -73$ TeV$^2$), since $m_{H_u}^2 - m_{H_d}^2$ is large and negative and $\mathrm{Tr} (m^2_q - m^2_l - 2 m^2_{u^c} + m^2_{d^c} + m^2_{e^c} )$ vanishes at the GUT scale. Inspection shows that the $X_t$ terms in (8) and (9) drive the stop and sbottom masses upwards, and the *S* terms in (8) and (10) drive the left-handed squark and right-handed slectron masses upwards. On the other hand, the *S* term in (9) drives the right-handed squark masses downwards. Since there are no counteracting *X* terms for the ${\tilde{u}_R}$ and ${\tilde{c}_R}$, these have lower masses than the other sfermions, opening the way to a ${\tilde{u}_R}/{\tilde{c}_R} - \tilde{\chi }^{0}_{1}$ coannihilation region.^12^

**Fig. 5:**
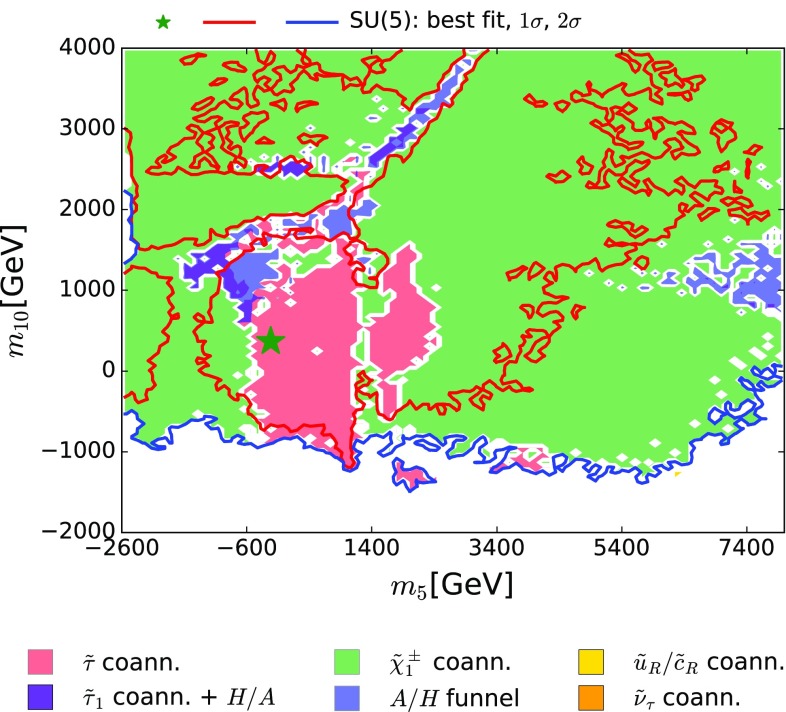
The $(m_5, m_{10})$ plane in the SUSY SU(5) GUT model. The *line colours* and *shadings* are the same as in Fig. 4

As discussed in more detail later, we used the Atom [144–148] simulation code for a dedicated verification that points in this region escape all the relevant LHC constraints. These points avoid exclusion by the LHC constraints through a combination of a strong mass degeneracy, $m_{{\tilde{u}_R}/{\tilde{c}_R}} - m_{\tilde{\chi }^{0}_{1}} \lesssim 50 \,\, \mathrm {GeV}$, leading to strong suppression of the standard  signature, and the reduction of the production rate compared to the simplified model that assumes mass degeneracy of all eight light flavour squarks (see Fig. 1). These effects are clearly visible in Fig. 18 of [4].

Figure 5 displays the corresponding information in the $(m_5, m_{10})$ plane of the SUSY SU(5) GUT model. As already reported in Table 3, here we see directly that the best-fit point has very small (and slightly negative) $m_5$, and that $m_{10}$ is somewhat larger, exploiting the possibility that $m_5 \ne m_{10}$, which is offered in this model. We also see again that the 68% CL region extends to values of $m_5$ and $m_{10}$ beyond the ${\tilde{\tau }}_1$ coannihilation region. We also note that in most of the rest of this plane $\tilde{\chi }^{\pm }_{1} - \tilde{\chi }^{0}_{1}$ coannihilation is dominant, with only scattered regions where rapid *H* / *A* annihilation is important, even in combination with ${\tilde{\tau }}_1$ coannihilation.

**Fig. 6:**
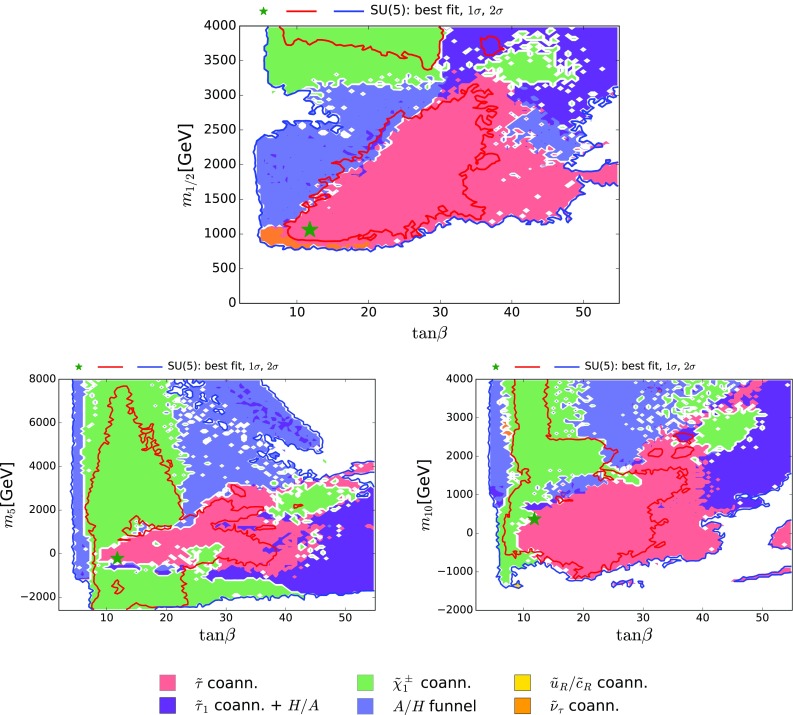
The $(\tan {\beta }, m_{1/2})$ plane (*upper panel*), the $(\tan {\beta }, m_{5})$ plane (*lower left panel*) and the $(\tan {\beta }, m_{10})$ plane (*lower right panel*) in the SUSY SU(5) GUT model. The *line colours* and *shadings* are the same as in Fig. 4

Projections of our results in the $(\tan {\beta }, m_{1/2}), (\tan {\beta }, m_5)$ and $(\tan {\beta }, m_{10})$ planes are shown in Fig. 6. We see that values of $\tan {\beta }\gtrsim 4$ are allowed at the 95% CL, that the range $\tan {\beta }\in (8, 57)$ is favoured at the 68% CL, and that there is no phenomenological upper limit on $\tan {\beta }$ at the 95% CL.^13^ The best-fit point has $\tan {\beta }= 13$, as also reported in Table 3.

The pink ${\tilde{\tau }}_1- \tilde{\chi }^{0}_{1}$ coannihilation region is very prominent in the $(\tan {\beta }, m_{1/2})$ projection shown in the upper panel of Fig. 6, as is the blue rapid *H* / *A* annihilation region and the purple ${\tilde{\tau }}_1- \tilde{\chi }^{0}_{1}$ coannihilation + *H* / *A* funnel hybrid region at large $\tan {\beta }$ and $m_{1/2}$. While the *H* / *A* funnel appears in the CMSSM only when $\tan \beta \gtrsim 45$ for $\mu > 0$ [18–22], in the SU(5) SUSY GUT model, it is found at significantly lower $\tan \beta$, due to the separation of $m_{H_u}$ and $m_{H_d}$ from $m_{5}$ and $m_{10}$, effectively making $m_A$ (and $\mu$) free parameters as in the NUHM2. There is also a region in the $(\tan {\beta }, m_{1/2})$ plane with $\tan {\beta }\lesssim 10$ and $m_{1/2} \sim 1000 \,\, \mathrm {GeV}$ where ${\tilde{\nu }_\tau }^\mathrm{NLSP}$ coannihilation is important.

The ${\tilde{\tau }}_1- \tilde{\chi }^{0}_{1}$ coannihilation region and the purple ${\tilde{\tau }}_1- \tilde{\chi }^{0}_{1}$ coannihilation $+$ *H* / *A* funnel hybrid region are prominent for $|m_5| \lesssim 3000 \,\, \mathrm {GeV}$ in the $(\tan {\beta }, m_5)$ and $(\tan {\beta }, m_{10})$ planes shown in the lower part of Fig. 6, with $\tilde{\chi }^{\pm }_{1} - \tilde{\chi }^{0}_{1}$ coannihilation dominant at smaller values of $\tan {\beta }$, in particular. The ${\tilde{u}_R}/{\tilde{c}_R} - \tilde{\chi }^{0}_{1}$ coannihilation region appears in a small island for $\tan {\beta }\sim 8$ and $m_{10} \sim -1200 \,\, \mathrm {GeV}$ in the $(\tan {\beta }, m_{10})$ plane shown in the lower right panel of Fig. 6.

**Fig. 7:**
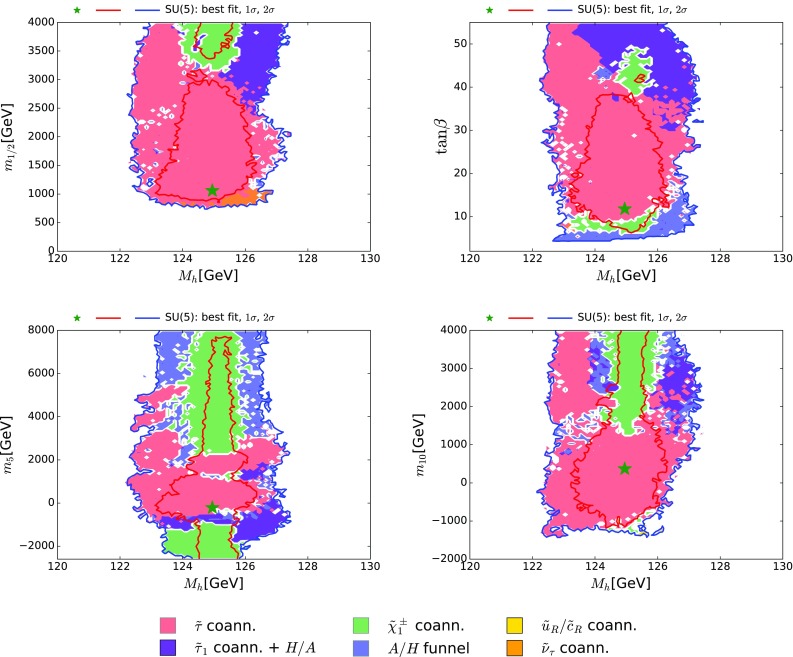
The $(M_h, m_{1/2})$ plane (*upper left panel*), the $(\tan {\beta }, M_h)$ plane (*upper right panel*), the $(M_h, m_5)$ plane (*lower left panel*) and the $(M_h, m_{10})$ plane (*lower right panel*) in the SUSY SU(5) GUT model. The *line colours* and *shadings* are the same as in Fig. 4

We display in Fig. 7 projections of our results for $M_h$ versus $m_{1/2}$ (upper left), $\tan {\beta }$ (upper right), $m_5$ (lower left) and $m_{10}$ (lower right). The predicted values of $M_h$ are well centred within the expected FeynHiggs uncertainty range around the value measured at the LHC, $M_h= 125.09 \pm 0.24 \,\, \mathrm {GeV}$ [116]. Moreover, the Dark Matter mechanisms do not exhibit any preference for values of $M_h$ above or below the nominal central value. Thus, there is no apparent tension between this LHC measurement and the other constraints on the SUSY SU(5) GUT model, with the notable exception of $(g-2)_\mu$.

As is well known, the calculation of $M_h$ in the MSSM is particularly sensitive to the value of the trilinear soft SUSY-breaking parameter $A_0$ as well as the stop squark masses. The latter depend in the SUSY SU(5) GUT model on $m_{10}$ and $m_{1/2}$, but are insensitive to $m_5$.

**Fig. 8:**
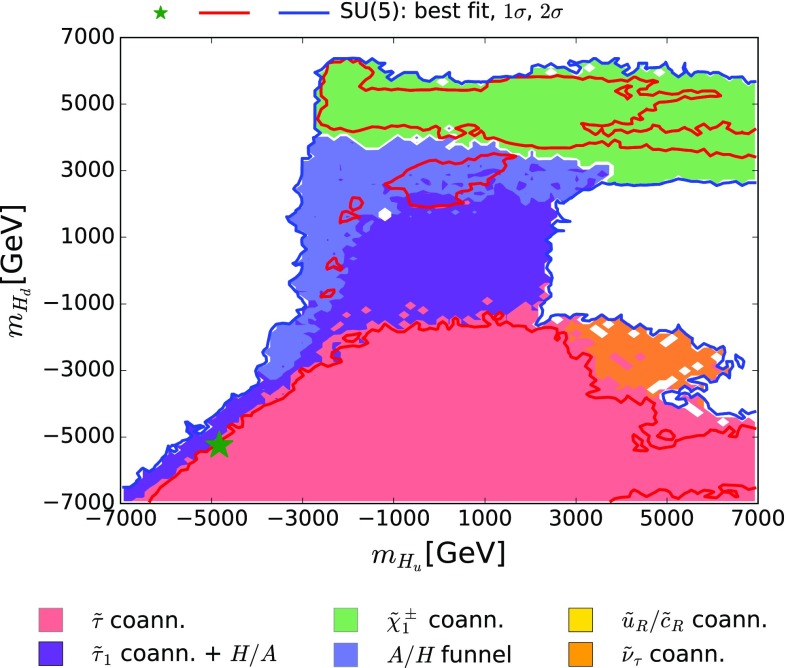
The $(m_{H_u}, m_{H_d})$ plane in the SUSY SU(5) GUT model. The *line colours* and *shadings* are the same as in Fig. 4

The $(m_{H_u}, m_{H_d})$ plane is shown in Fig. 8. We see that the best-fit point lies in the quadrant where both $m_{H_u}$ and $m_{H_d}$ are negative, and that the 68% CL region extends also to the quadrant where $m_{H_d}$ is negative and $m_{H_u}$ is positive, as does the ${\tilde{\tau }}_1- \tilde{\chi }^{0}_{1}$ coannihilation region. On the other hand, the $\tilde{\chi }^{\pm }_{1} - \tilde{\chi }^{0}_{1}$ coannihilation region lies in the upper quadrants where $m_{H_d} > 0$. There is also an intermediate region, characterized by the H/A funnel mechanism and its hybridization with ${\tilde{\tau }}_1$ coannihilation, part of which is also allowed at the 68% CL. There is also a region with $M_{H_u} \sim 4000 \,\, \mathrm {GeV}, M_{H_d} \sim -3000 \,\, \mathrm {GeV}$ where ${\tilde{\nu }_\tau }^\mathrm{NLSP}$ coannihilation is important.

**Fig. 9:**
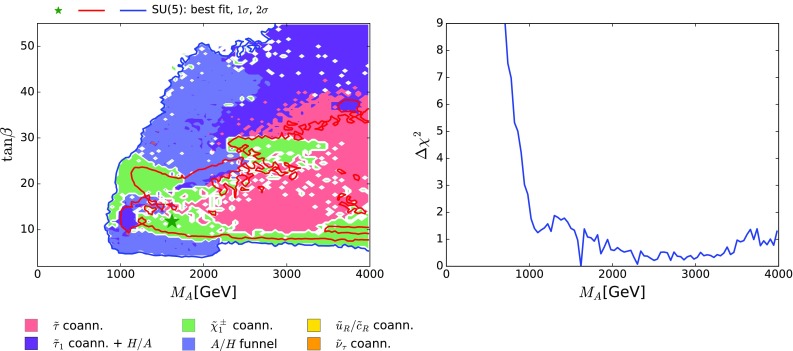
*On the left* the $(M_A, \tan \beta )$ plane in the SUSY SU(5) GUT model. The *line colours* and *shadings* are the same as in Fig. 4. *On the right* the $\chi ^2$ likelihood function for the pseudoscalar mass

The left panel in Fig. 9 displays the $(M_A, \tan {\beta })$ plane in the supersymmetric SU(5) GUT model. We see that $M_A\gtrsim 800 (1000) \,\, \mathrm {GeV}$ at the $\Delta \chi ^2 = 5.99~(2.30)$ level, corresponding to the 95 (68) % CL, which is largely due to the interplay of the indirect constraints on $(M_A, \tan {\beta })$ such as $M_h$ (see also [135]) as well as the direct constraints from the LHC heavy MSSM Higgs searches. Even for large $\tan {\beta }$, where these constraints impose the strongest lower limit on $M_A$, it is much weaker than our global limit, which is $M_A\gtrsim 2800 ({>} 4000) \,\, \mathrm {GeV}$ at the 95 (68) % CL.^14^ We observe the same behaviour in the right panel of Fig. 9, where the one-dimensional likelihood profile for $M_A$ is shown. Indeed, the lightest pseudoscalar mass allowed at $\Delta \chi ^2 = 4$ is ${\sim }920$ GeV. The best-fit point in the global fit has $(M_A, \tan {\beta }) \simeq (1600 \,\, \mathrm {GeV}, 13)$: this is considerably beyond the present and projected LHC reach, though poorly determined.

## One-dimensional likelihood functions

We now discuss the one-dimensional $\Delta \chi ^2$ functions for various observable quantities.

**Fig. 10:**
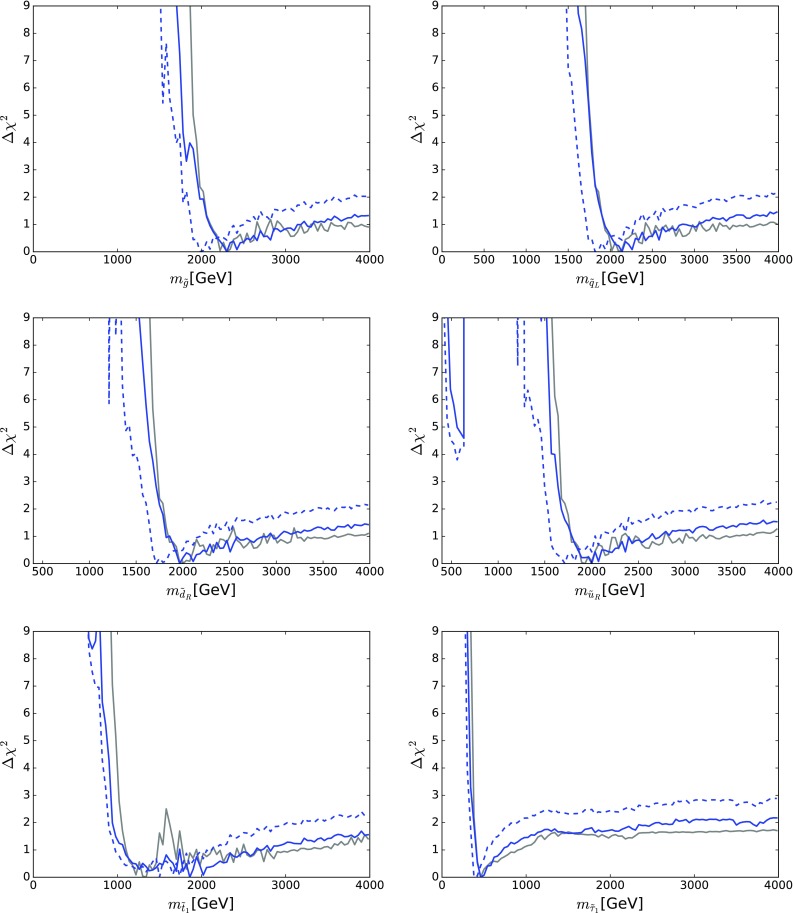
The $\chi ^2$ likelihood functions in the SUSY SU(5) GUT model (*blue lines*) for the gluino mass (*top left panel*), the left-handed squark mass (*top right panel*), the right-handed down squark mass (*centre left panel*), the right-handed up squark mass (*centre right panel*), the lighter stop squark mass (*lower left panel*) and the lighter stau slepton mass (*lower right panel*). The *dashed blue lines* show the result of omitting the LHC 13-TeV constraints, and the *grey lines* represent ‘fake’ NUHM2 results obtained by selecting a subset of the SU(5) sample with $m_5/m_{10} \in [0.9, 1.1]$

Figure 10 displays those for $m_{\tilde{g}}$ (top left), $m_{\tilde{q}_L}$ (top right), $m_{\tilde{d}_R}$ (centre left), $m_{\tilde{u}_R}$ (centre right), $m_{\tilde{t}_1}$ (bottom left) and $m_{{\tilde{\tau }}_1}$ (bottom right). The solid blue line in each panel corresponds to the current analysis of the supersymmetric SU(5) model including LHC Run 2 data at 13 TeV, the dashed blue line shows the result of an SU(5) fit in which the LHC 13-TeV results are not included, and the solid grey line corresponds to ‘fake’ NUHM2-like results obtained by selecting a subset of the SU(5) sample with $m_5/m_{10} \in [0.9, 1.1]$, which we discuss in more detail later.^15^

**Fig. 11:**
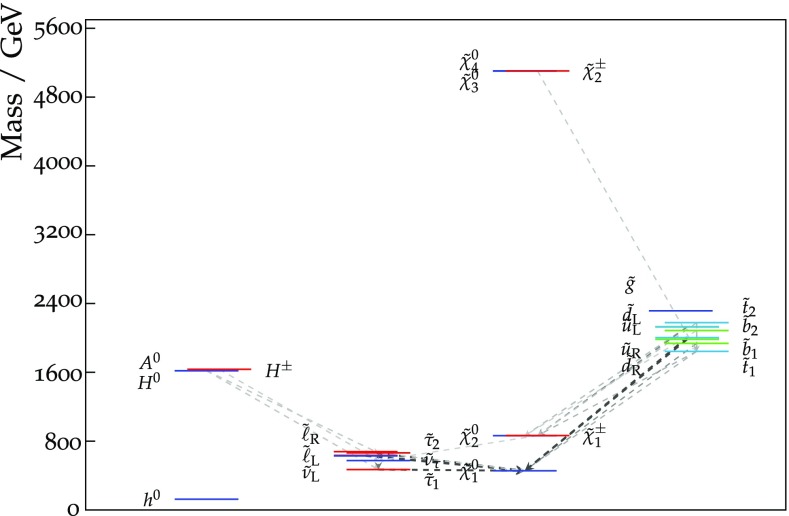
The spectrum at the best-fit point in the SUSY SU(5) GUT model. Decay branching ratios (BRs) exceeding 20% are denoted by *dashed lines*, which are thicker for more important BRs

**Fig. 12:**
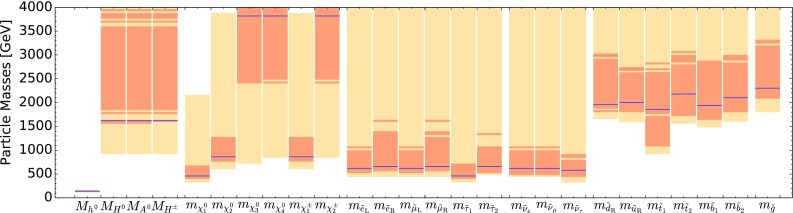
The one-dimensional 68 and 95% CL ranges of masses we obtain for the current fit in the supersymmetric SU(5) model, shown in dark and light orange, respectively. The best-fit point is represented by blue lines

The current SU(5) fit exhibits minima of $\chi ^2$ at masses ${\lesssim } 2.5 \,\, \mathrm {TeV}$: $m_{\tilde{g}}\simeq 2600 \,\, \mathrm {GeV}$, common squark mass $m_{\tilde{q}}\simeq 2200 \,\, \mathrm {GeV}$, $m_{\tilde{u}_R}$, $m_{\tilde{d}_R}$, $m_{\tilde{t}_1}\simeq 2200 \,\, \mathrm {GeV}$ and $m_{{\tilde{\tau }}_1}\simeq 540 \,\, \mathrm {GeV}$, followed by a rise at higher mass towards a plateau with $\Delta \chi ^2 \lesssim 2$. The minimum is relatively sharp for $m_{\tilde{g}}$, $m_{\tilde{q}}$ and $m_{{\tilde{\tau }}_1}$, whereas it is broader for $m_{\tilde{t}_1}$. The exact values are listed in Table 4 and depicted in Fig. 11. In this figure we also indicate decay branching ratios (BRs) exceeding 20% by dashed lines, which are thicker for more important BRs. Figure 12 displays the one-dimensional 68 and 95% CL ranges for the Higgs and sparticle masses in the supersymmetric SU(5) model as darker and lighter coloured bands, with the best-fit values shown as blue lines.

Concerning future $e^+e^-$ colliders, one can see that the best-fit masses of the lightest neutralino and stau are $\sim 500 \,\, \mathrm {GeV}$, and some other 68% CL ranges go down to $500 \,\, \mathrm {GeV}$, offering the possibility of pair production at a collider with $\sqrt{s} \sim 1 \,\, \mathrm {TeV}$, as envisaged for the final stage of the ILC [172, 173]. Going to higher centre-of-mass energies, e.g., $\sqrt{s} \lesssim 3 \,\, \mathrm {TeV}$ [173, 174] as anticipated for CLIC, significant fractions of the 68% CL ranges of electroweak sparticle masses can be covered.

As already noted, a novel feature of the SUSY SU(5) GUT model with ($m_5 \ne m_{10}$) is that the ${\tilde{u}_R}$ and ${\tilde{c}_R}$ may be much lighter than the other squarks. This leads to the possibility of a ${\tilde{u}_R}/{\tilde{c}_R} - \tilde{\chi }^{0}_{1}$ coannihilation strip where $m_{\tilde{u}_R}$ and $m_{\tilde{c}_R} \sim 500 \,\, \mathrm {GeV}$, which is visible as a second local minimum of $\chi ^2$ with $\Delta \chi ^2 > 4$ in the centre right panel of Fig. 10.

**Fig. 13:**
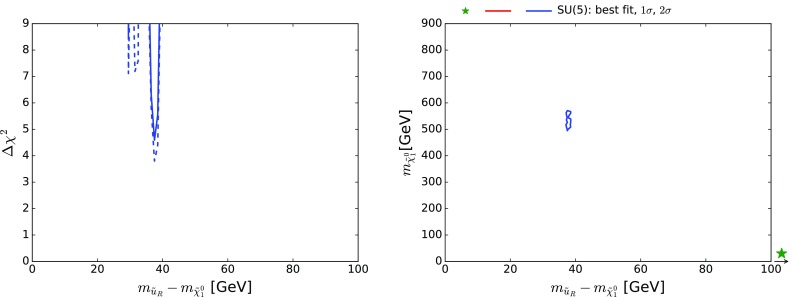
*Left panel* the $\chi ^2$ likelihood function in the SUSY SU(5) GUT model for $m_{\tilde{u}_R} - m_{\tilde{\chi }^{0}_{1}}$ in the ${\tilde{u}_R}/{\tilde{c}_R} - \tilde{\chi }^{0}_{1}$ coannihilation strip region (the *solid*/*dashed line* includes/omits the 13-TeV LHC data). *Right panel* the region of the $(m_{\tilde{u}_R}, m_{\tilde{\chi }^{0}_{1}})$ plane where $\Delta \chi ^2 < {5.99}$

We have checked specifically whether this strip is allowed by the available LHC constraints. To this end, we verified using the Atom simulation code that points along this strip are consistent with the published constraints from the LHC 8-TeV data. We have also checked that this strip is consistent with the preliminary simplified-model search for $\tilde{q}\tilde{q}+ \tilde{q}\bar{\tilde{q}}$ at 13 TeV reported by CMS. The left panel of Fig. 13 displays as a solid/dashed blue line the one-dimensional $\chi ^2$ function for $m_{\tilde{u}_R} - m_{\tilde{\chi }^{0}_{1}}$ including/omitting the 13-TeV data (the corresponding lines for $m_{\tilde{c}_R} - m_{\tilde{\chi }^{0}_{1}}$ are very similar), and the right panel of Fig. 13 shows the region of the $(m_{\tilde{u}_R}, m_{\tilde{\chi }^{0}_{1}})$ plane where $\Delta \chi ^2 < {5.99}$, i.e., allowed at the 95% CL. We find that $\sigma (\tilde{q}\tilde{q}+ \tilde{q}\bar{\tilde{q}}) < 0.1$ pb in this region, whereas the cross-section upper limit as given in [9] is ${\gtrsim } 1$ pb. We conclude that this simplified-model search does not affect the likelihood in this ${\tilde{u}_R}/{\tilde{c}_R} - \tilde{\chi }^{0}_{1}$ coannihilation strip region. However, it will be explored further by future LHC data with increased luminosity.

Finally, we comment on the impact of the constraints from mono-jet searches [175–177]. These searches are designed to be sensitive to the highly compressed mass region by limiting the multiplicity of the high $p_T$ jets. In the ${\tilde{u}_R}/{\tilde{c}_R} - \tilde{\chi }^{0}_{1}$ coannihilation region, the mass difference is mildly compressed, $m_{\tilde{u}_R/\tilde{c}_R} - m_{\tilde{\chi }^{0}_{1}} \sim 40$ GeV, and the jets from ${\tilde{u}_R}/{\tilde{c}_R}$ decays are still resolvable from the background. Such extra jets will spoil the characteristic of the mono-jet event and reduce the efficiency. The degradation of the sensitivity for the mildly compressed region is clearly seen for example in Fig. 5 of [175]. For this reason, the mono-jet searches lose sensitivity to the ${\tilde{u}_R}/{\tilde{c}_R} - \tilde{\chi }^{0}_{1}$ coannihilation region, compared to the jets +  analysis [9], and we do not consider them in this paper.

**Table 4:** Particle masses at the best-fit point in the SUSY SU(5) GUT model (in GeV units)

${{\tilde{\tau }}_1}$	${{\tilde{\tau }}_2}$	${\tilde{e}_L}$	${\tilde{e}_R}$	${{\tilde{\nu }}_\tau }$	${\tilde{q}_L}$	${\tilde{t}_1}$	${\tilde{t}_2}$
470	660	630	678	570	2130	1840	2180
${\tilde{b}_1}$	${\tilde{b}_2}$	${\tilde{u}_R}$	${\tilde{d}_R}$	${\tilde{g}}$	$M_{H,A}$	$m_{\tilde{\chi }^{0}_{1}}$	$m_{\tilde{\chi }^{0}_{2},\tilde{\chi }^{\pm }_{1}}$
1940	2090	2000	1980	2310	1620	460	860

Another novel feature of the SUSY SU(5) GUT model is visible in Table 4 and Fig. 11. Having $m_5 \ne m_{10}$ allows the possibility of strong mixing between the ${{\tilde{\tau }}_R}$ in the $\mathbf {10}$ representation and the ${{\tilde{\tau }}_L}$ in the ${\bar{\mathbf{5}}}$ representation. For example, at the best-fit point the ${{\tilde{\tau }}_1}$ is an almost equal mixture of $\tilde{\tau }_{L}$ and $\tilde{\tau }_{R}$:

15 $$\begin{aligned} \tilde{\tau }_{1} = 0.70\, \tilde{\tau }_{L} + 0.72\, \tilde{\tau }_{R}. \end{aligned}$$

This large mixing explains the level repulsion $\Delta m \simeq 200 \,\, \mathrm {GeV}$ between the ${{\tilde{\tau }}_1}$ and ${{\tilde{\tau }}_2}$ seen in Table 4, which is much larger than the splitting $\Delta m \simeq 50 \,\, \mathrm {GeV}$ between the almost unmixed ${\tilde{e}_1} \sim {\tilde{e}_R}$ and ${\tilde{e}_2} \sim {\tilde{e}_L}$, which is also seen in Table 4.

**Fig. 14:**
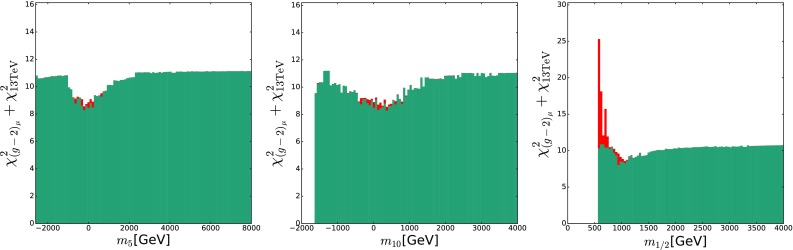
The $\chi ^2$ contributions of $(g-2)_\mu$ (*teal*) and LHC 13-TeV data (*red*) in the SUSY SU(5) GUT model, as functions of $m_5$ (*left panel*), $m_{10}$ (*middle panel*) and $m_{1/2}$ (*right panel*)

We show in Fig. 14 the contribution to the global $\chi ^2$ function of $(g-2)_\mu$ (in teal), as a function of $m_5$ (left panel), $m_{10}$ (middle panel) and $m_{1/2}$ (right panel). In each case, there is a well-defined minimum that is lower than the plateau at large mass values by $\Delta \chi ^2 \gtrsim 2$. In contrast, the contributions to the global $\chi ^2$ function of the other observables are relatively featureless over large ranges of $m_5$, $m_{10}$ and $m_{1/2}$, with the exception of the contribution from the LHC 13-TeV data (mainly due to the  constraint), which rises sharply at low $m_{1/2}$, as shown in the stacked red histogram in the right panel of Fig. 14. Because we profile over the other parameters, this does not have much impact on the dependence of $\chi ^2$ on $m_5$ and $m_{10}$, as seen in the left and middle panels. The well-defined minima seen in the $(g-2)_\mu$ contributions in the left and middle panels of Fig. 14 occur at quite small values of $m_5$ and $m_{10}$, reflecting the fact that $(g-2)_\mu$ is sensitive to the soft symmetry-breaking contributions to the masses of both ${{\tilde{\mu }}_L}$ and ${{\tilde{\mu }}_{R}}$. These are $m_5$ and $m_{10}$, respectively, so maximizing the SUSY contribution to $(g-2)_\mu$ and thereby minimizing the $(g-2)_\mu$ contribution to $\chi ^2$ prefers small values of both $m_5$ and $m_{10}$. Similarly, the SUSY contribution to $(g-2)_\mu$ is suppressed for large gaugino masses, explaining the aversion to large $m_{1/2}$ seen in the right panel of Fig. 14.

**Fig. 15:**
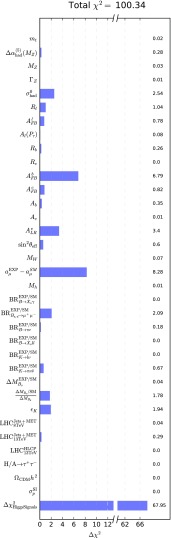
The $\chi ^2$ pulls for different observables at the best-fit point in the SUSY SU(5) model

The principal contributions to the global $\chi ^2$ function at the best-fit point for the SUSY SU(5) GUT model are given in Table 5, and the corresponding pulls at the best-fit point are displayed graphically in Fig. 15. Apart from $(g-2)_\mu$, the other contributions deserving of comment include the following. The large contribution from HiggsSignals reflects the large number of channels considered, and has negligible variation for most of the points in our sample. We note that $A_\mathrm{FB} (b)$ makes a contribution that is not much smaller than that of $(g-2)_\mu$ at the best-fit point, and that $A_\mathrm{LR}^{e}$ and $\sigma ^0_\mathrm{had}$ also make relatively large contributions to the global $\chi ^2$ function. These observables reflect the residual tensions in the electroweak precision observables at the *Z* peak, which are present in the SM and the SUSY SU(5) GUT model is unable to mitigate.

**Table 5:** The principal $\chi ^2$ contributions of observables at the best-fit point in the SUSY SU(5) GUT model, together with the total $\chi ^2$ function

$A_\mathrm{LR}^{e}$	$A_b$	$A_\mathrm{FB}(\ell )$	$A_\mathrm{FB}(b)$	$A_\mathrm{FB}(c)$	$A_{l}(P_\tau )$	
3.40	0.35	0.78	6.79	0.82	0.08	
$R_b$	BR($b \rightarrow s \gamma$)	BR($B_u \rightarrow \tau \nu _\tau$)	$\Omega _{\tilde{\chi }^{0}_{1}} h^2$	$\sigma ^\mathrm{SI}_p$	$\mathrm{BR}(B_{s, d} \rightarrow \mu ^{+}\mu ^{-})$	
0.26	0.00	0.18	0.00	0.00	2.09	
$\sin ^2\theta _{\mathrm {eff}}$	$M_W$	$R_l$	$R(K \rightarrow l \nu )$	$(g-2)_\mu$	$M_h$	
0.60	0.07	1.04	0.0	8.28	0.01	
$\sigma _\mathrm{had}^0$	${\frac{\Delta M_{B_s}}{\Delta M_{B_d}}}$	$\epsilon _K$	$H/A \rightarrow \tau ^+ \tau ^-$	HiggsSignals	LHC	Total
2.54	1.78	1.94	0.00	67.95	0.3	100.34

In order to compare the quality of the SU(5) fit to the results of previous MasterCode analyses of competing models [15], we follow the prescription used there of subtracting from the total $\chi ^2$ given in Table 5 and Fig. 15, namely 100.34, the $\chi ^2$ contributions originating from HiggsSignals [136, 137], which dominate the global $\chi ^2$ function and would bias the analysis. Fig. 15 lists 36 separate contributions to the total $\chi ^2$ function. The first three ($m_t, M_Z$, and $\Delta \alpha _\mathrm{had}^{(5)} (M_Z)$) are treated as nuisance parameters and the two LHC MET constraints at 8 and 13 TeV are applied as a single constraint. Omitting the HiggsSignals constraints in our determination of the number of degrees of freedom leaves 30 constraints, with seven parameters for the SU(5) model and hence 23 degrees of freedom.The $\chi ^2$ contributions from the relevant constraints sum to 32.39, corresponding to a $\chi ^2$ probability of 9%. This can be compared with the $\chi ^2$ probability values of 11, 12, 11 and 31% found in [15] for the CMSSM, NUHM1, NUHM2 and pMSSM10, respectively, using LHC Run 1 constraints. However, as in [15], we stress that these $\chi ^2$ probabilities are only approximate since, for example, they neglect correlations between the observables. A more complete treatment using toys, as done in the last reference of [37–45], is beyond the scope of this work.

There are a couple of important corollaries to this observation, one concerning $m_{\tilde{t}_1}$. It is sensitive to $A_0$ as well as the soft SUSY-breaking contributions to the ${\tilde{t}_L}$ and ${\tilde{t}_R}$ mass parameters (which are both given by $m_{10}$ in the SUSY SU(5) GUT model). Since $A_0$ is relatively poorly determined, the $\chi ^2$ minimum for $m_{\tilde{t}_1}$ is relatively shallow, as seen in the lower left panel of Fig. 10.

The second observation concerns the sign of $\mu$. All our analysis has been for $\mu > 0$, which is the sign capable of mitigating the discrepancy between the experimental value of $(g-2)_\mu$ and the SM prediction. For $\mu < 0$, the large-mass plateau would have a similar height as in Fig. 14, but the $\chi ^2$ function would rise monotonically at low values of $m_5$, $m_{10}$ and $m_{1/2}$, instead of featuring a dip. Thus, the $\mu < 0$ possibility would be disfavoured by $\Delta \chi ^2 \gtrsim 2$, and the global minimum would lie at large masses and be ill defined.

**Fig. 16:**
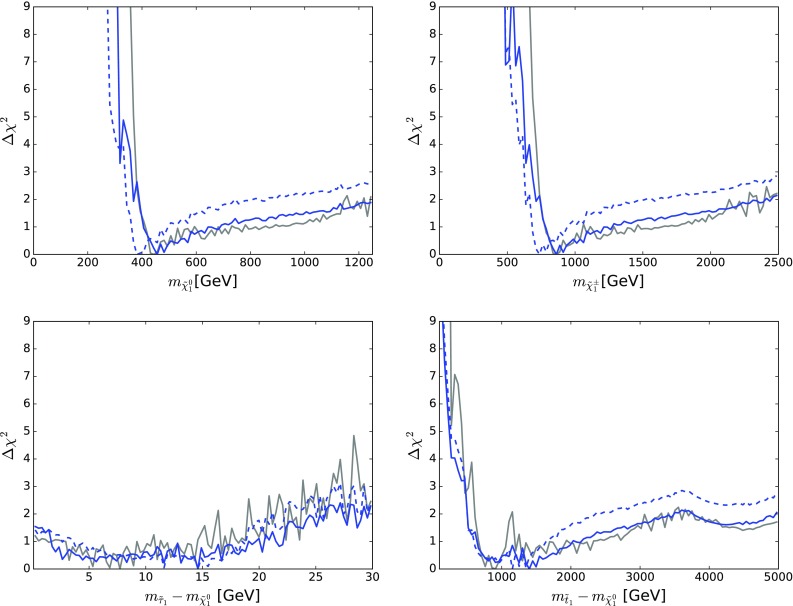
The $\chi ^2$ likelihood functions in the SUSY SU(5) GUT model for the $\tilde{\chi }^{0}_{1}$ mass (*upper left panel*), the $\tilde{\chi }^{\pm }_{1}$ mass (*upper right panel*), the ${{\tilde{\tau }}_1} -\tilde{\chi }^{0}_{1}$ mass difference (*lower left panel*) and the ${\tilde{t}_1} - \tilde{\chi }^{0}_{1}$ mass difference (*lower right panel*). The *dashed blue lines* shows the result of omitting the LHC 13-TeV constraints, and the *grey lines* represent ‘fake’ NUHM2 results obtained by selecting a subset of the SU(5) sample with $m_5/m_{10} \in [0.9, 1.1]$

The $\chi ^2$ distributions for some more observables are shown in Fig. 16, We see that the minima for $m_{\tilde{\chi }^{0}_{1}}$ (upper left panel) and $m_{\tilde{\chi }^{\pm }_{1}}$ (upper right panel) are quite well defined, mirroring the structure in the $\chi ^2$ function for $m_{{\tilde{\tau }}_1}$ shown in the lower right panel of Fig. 10. The preference for a (very) small ${\tilde{\tau }_1} - \tilde{\chi }^{0}_{1}$ mass difference is seen in the lower left panel of Fig. 16, and reflects the fact, commented on in connection with many previous figures, that the best-fit point and much of the 68% CL region lies in the ${{\tilde{\tau }}_1} - \tilde{\chi }^{0}_{1}$ coannihilation region. On the other hand, a small $m_{\tilde{t}_1}- m_{\tilde{\chi }^{0}_{1}}$ mass difference is disfavoured, as seen in the lower right plot of Fig. 16, reflecting the fact that stop coannihiliation does not play a significant role.

**Fig. 17:**
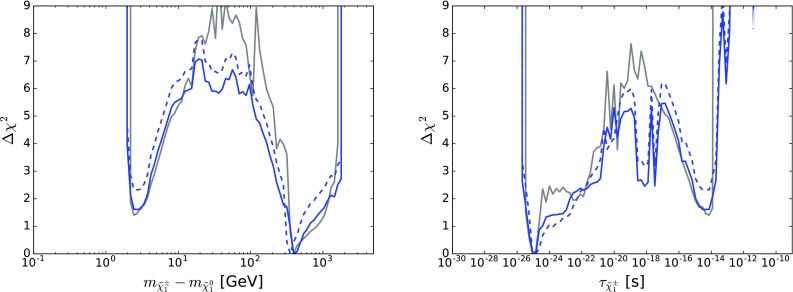
The $\chi ^2$ likelihood functions in the SUSY SU(5) GUT model for the $\tilde{\chi }^{\pm }_{1} - \tilde{\chi }^{0}_{1}$ mass (*left panel*) and the $\tilde{\chi }^{\pm }_{1}$ lifetime (*right panel*). The *dashed blue lines* shows the result of omitting the LHC 13-TeV constraints, and the *grey lines* represent ‘fake’ NUHM2 results obtained by selecting a subset of the SU(5) sample with $m_5/m_{10} \in [0.9, 1.1]$

The $\tilde{\chi }^{\pm }_{1} - \tilde{\chi }^{0}_{1}$ coannihilation region is prominent in the previous figures, and also contains parameter sets that are preferred at the 68% CL. Hence a small $\tilde{\chi }^{\pm }_{1} - \tilde{\chi }^{0}_{1}$ mass difference is also allowed at the $\Delta \chi ^2 \gtrsim 1$ level, as seen in the left panel of Fig. 17, although the best-fit point has $m_{\tilde{\chi }^{\pm }_{1}} - m_{\tilde{\chi }^{0}_{1}} \sim 470 \,\, \mathrm {GeV}$. However, values of the $\tilde{\chi }^{\pm }_{1}$ lifetime that are allowed at the 95% CL are all too short to provide a long-lived particle signal, as seen in the right panel of Fig. 17.^16^

**Fig. 18:**
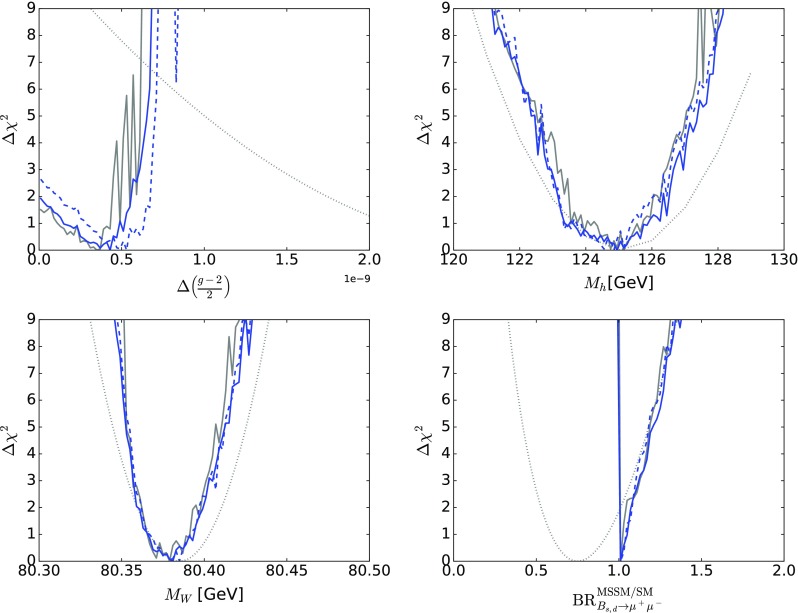
The $\chi ^2$ likelihood functions in the SUSY SU(5) GUT model for $(g-2)_\mu /2$ (*upper left panel*), $M_h$ (*upper right panel*), $M_W$ (*lower left panel*), and $\mathrm{BR}(B_{s, d} \rightarrow \mu ^{+}\mu ^{-})$ (*lower right panel*). The *dashed blue lines* shows the result of omitting the LHC 13-TeV constraints, and the *solid grey lines* represent ‘fake’ NUHM2 results obtained by selecting a subset of the SU(5) sample with $m_5/m_{10} \in [0.9, 1.1]$., and the *dotted grey lines* represent the current experimental measurements with their uncertainties

We now discuss the one-dimensional likelihood functions for electroweak precision observables and observables in the flavour sector shown in Fig. 18, together with the current experimental measurements and their uncertainties shown as dotted grey lines. The upper left panel displays $(g-2)_\mu$, and we see that the global minimum occurs for $\Delta (g-2)_\mu \simeq 0.4 \times 10^{-9}$, with $\Delta \chi ^2 \lesssim - 2$ compared to the case $\Delta (g-2)_\mu = 0$. We see again that the SUSY SU(5) GUT model is able to mitigate slightly the discrepancy between the SM and the measurement of $(g-2)_\mu$, although it does not provide a substantial improvement over the SM prediction.

As for $M_h$, as shown in the upper right panel of Fig. 18 the $\chi ^2$ function is minimized close to the nominal experimental value, and is quite symmetric, showing no indication of any tension in the SUSY SU(5) GUT model fit. Likewise, the best-fit value of $M_W$ (lower left panel of Fig. 18) is highly compatible with the experimental measurement, and that for $\mathrm{BR}(B_{s, d} \rightarrow \mu ^{+}\mu ^{-})$ (lower right panel) is very close to the SM prediction, and hence also compatible with the experimental measurement. We note that, whereas values of $\mathrm{BR}(B_{s, d} \rightarrow \mu ^{+}\mu ^{-})$ that are slightly larger than the SM value are possible, smaller values are strongly disfavoured in the SUSY SU(5) GUT model.

**Fig. 19:**
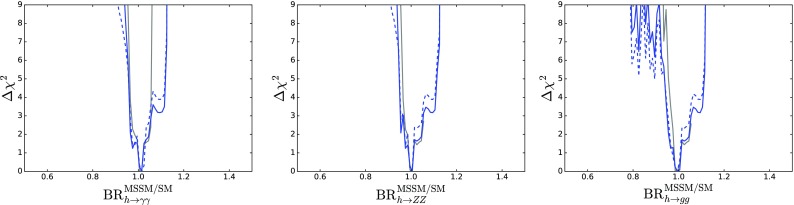
The $\chi ^2$ likelihood functions for the ratios of the SUSY SU(5) and SM predictions for the BRs of $h \rightarrow \gamma \gamma$ (*left panel*), $h \rightarrow Z Z^*$ (*middle panel*) and $h \rightarrow gg$ decays (*right panel*). The *dashed blue lines* shows the result of omitting the LHC 13-TeV constraints, and the *grey lines* represent ‘fake’ NUHM2 results obtained by selecting a subset of the SU(5) sample with $m_5/m_{10} \in [0.9, 1.1]$

## Higgs branching ratios

We present in Fig. 19 the one-dimensional likelihood functions for the ratios of supersymmetric SU(5) and SM predictions for the BRs of $h \rightarrow \gamma \gamma$ (left panel), $h \rightarrow Z Z^*$ (middle panel)^17^ and $h \rightarrow gg$ decays (right panel). We see that in each case the preferred region in the fit corresponds to a prediction in the SU(5) model that deviates from the SM case by at most a few %, whereas the present experimental uncertainties in the different coupling modifiers squared (employing some theory assumptions) are typically $\mathcal{O}(30)$% [179], and a precision of ${\mathcal O}(5-10\%)$ (with the same theory assumptions) can be reached by the end of the LHC programme. On the other hand, future $e^+e^-$ colliders such as the ILC, CLIC or FCC-ee anticipate a precision at the percent level for couplings to fermions and at the permille level for couplings to massive gauge bosons [173, 180]. This offers the possiblity that deviations from the SM in the SUSY SU(5) GUT model can be measured in the future.

## Comparison with previous results

In previous papers we have studied the CMSSM, NUHM1 and NUHM2 using the LHC 8-TeV results and earlier DM scattering constraints. None of these models are directly comparable to the supersymmetric SU(5) model studied here, which has four different soft SUSY-beaking scalar mass parameters, $m_5, m_{10}, m_{H_u}$ and $m_{H_d}$. The most similar is the NUHM2, which has the three parameters $m_0 = m_5 = m_{10}, m_{H_u}$ and $m_{H_d}$. Here we compare the supersymmetric SU(5) results found in this paper using LHC 13-TeV data with ‘fake’ NUHM2 results obtained by selecting a subset of this SU(5) sample with $m_5/m_{10} \in [0.9, 1.1]$ (which were also displayed as grey lines in Fig. 10) and with previous NUHM2 results [14].

**Fig. 20:**
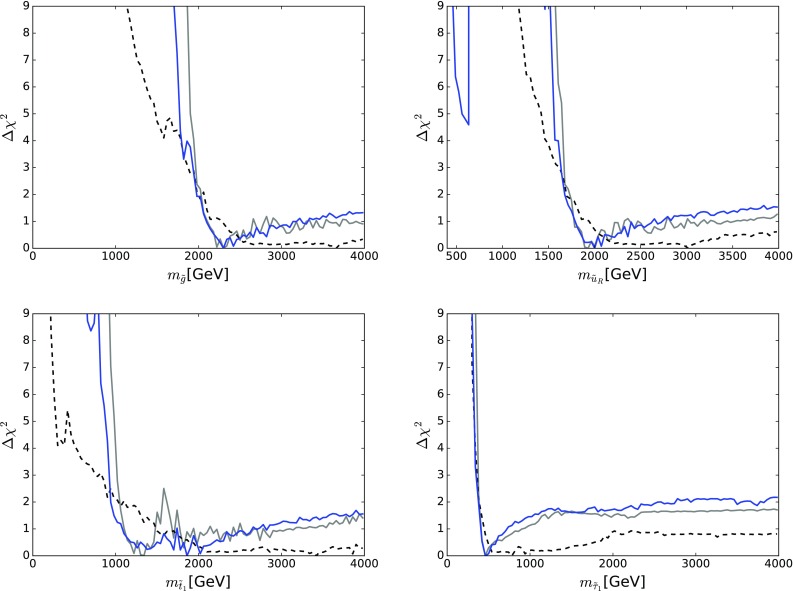
The one-dimensional $\chi ^2$ likelihood functions for the full SU(5) sample (*solid blue lines*) and in the restriction of the SUSY SU(5) GUT model sample to $m_5/m_{10} \in [0.9, 1.1]$ (*solid grey lines*) compared to those in our previous NUHM2 analysis [14] (*dashed grey lines*) for $m_{\tilde{g}}$ (*upper left panel*), $m_{\tilde{q}}$ (*upper right panel*), $m_{\tilde{t}_1}$ (*lower left panel*), and $m_{{\tilde{\tau }}_1}$ (*lower right panel*)

Figure 20 compares the one-dimensional $\chi ^2$ likelihood functions for $m_{\tilde{g}}$ (upper left), $m_{\tilde{q}_R}$ (upper right), $m_{\tilde{t}_1}$ (lower left) and $m_{{\tilde{\tau }}_1}$ (lower right) found in the SU(5) model including LHC 13-TeV constraints (solid blue lines) with the restricted fake NUHM2 sample (solid grey lines) and, for comparison, results from our previous NUHM2 analysis that used only the LHC 7- and 8-TeV constraints (dashed grey lines) [14]. We see here and in Fig. 10 that the restricted ‘fake’ NUHM2 sample exhibits, in general, best-fit masses that are similar to those found in the full SU(5) sample. The most noticeable differences are that lower masses are disfavoured in the restricted sample relative those in the full SU(5) model, indicating that the latter has some limited ability to relax the NUHM2 lower bounds on sparticle masses, e.g., at the 95% CL. The previous NUHM2 analysis [14] also yielded similar best-fit masses but, as could be expected, gave 95% CL lower limits on sparticle masses that were further relaxed. Similar features can also be observed in Figs. 16, 17, 18 and 19, where we have also included the ‘fake’ NUHM2 subsample.

Restricting further our SU(5) to mimic the NUHM1, let alone the CMSSM, is not useful because of the increased sampling uncertainties in such restricted samples. However, we showed in [14] that our NUHM2 LHC 7- and 8-TeV results for the exhibited sparticle masses were broadly similar to those for the NUHM1 and the CMSSM [13], and we expect the impacts of the LHC 13-TeV data on these models to be comparable to that in the NUHM2.

Finally, we ask whether or not there is a significant improvement in the SU(5) fit compared to that in the NUHM2 subsample, thanks to the additional parameter ($m_5$ and $m_{10}$ replacing $m_0$). The NUHM2 subsample has a total $\chi ^2 = 100.8$, which is reduced to 32.8 when we remove the contributions from HiggsSignals, as discussed earlier. It should be noted that the NUHM2 subsample is statistically significantly smaller than that of the SU(5) sample. The quoted NUHM2 $\chi ^2$ represents only an upper bound on the $\chi ^2$ of the best-fit point that would be found in a more complete sample of the NUHM2. Since the NUHM2 model has one less parameter than the SU(5) model, it has 24 degrees of freedom, and its $\chi ^2$ probability is 11%. According to the Wilks test [181], the probability that the data are represented better by the SU(5) model than by the NUHM2 subsample is 50%, while the *F* test [182] yields a 40% probability. Therefore we conclude that there is no evidence that the extra parameter of SU(5) provides a significant improvement.

## The possibility of a long-lived ${\varvec{{\tilde{\tau }}_1}}$

**Fig. 21:**
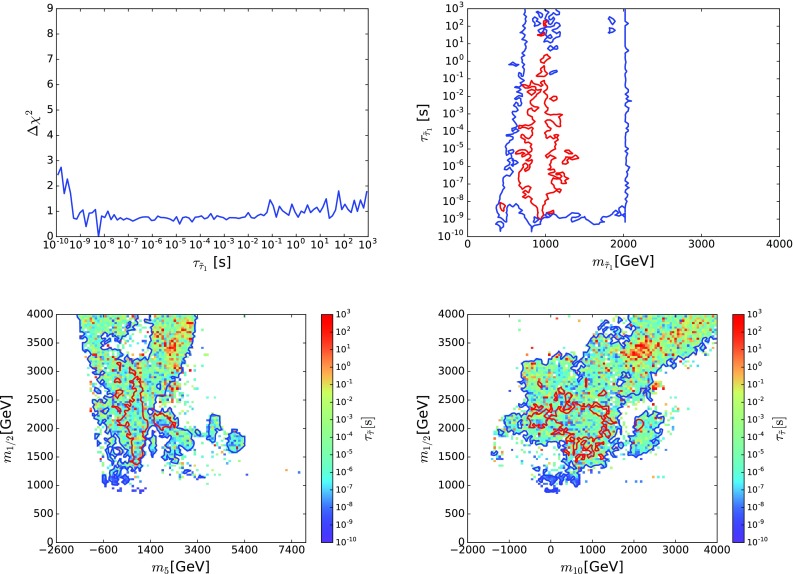
*Upper left panel* the global $\chi ^2$ function in the SUSY SU(5) GUT model as a function of the $\tilde{\tau }_1$ lifetime. *Upper right panel* the $(m_{{\tilde{\tau }}_1}, \tau _{\tilde{\tau }_1})$ plane, shaded according to the values of $\tau _{\tilde{\tau }_1}$, as indicated. *Lower panels* the $(m_5, m_{1/2})$ and $(m_{10}, m_{1/2})$ planes, *coloured* according to the values of $\tau _{{\tilde{\tau }}_1}$. The 68 and 95% CL contours in these three planes are *coloured red* and *blue*, respectively

The possibility of a very small ${{\tilde{\tau }}_1} - \tilde{\chi }^{0}_{1}$ mass difference opens up the possibility that the ${{\tilde{\tau }}_1}$ might have a long lifetime, as discussed in the contexts of the CMSSM, NUHM1 and NUHM2 in [16]. This would occur if $m_{\tilde{\tau }_1} - m_{\tilde{\chi }^{0}_{1}} < m_\tau$. As seen in the lower left panel of Fig. 16, the best-fit point has a mass difference ${\sim } 20 \,\, \mathrm {GeV}$, outside this range, but $m_{\tilde{\tau }_1} - m_{\tilde{\chi }^{0}_{1}} < m_\tau$ is allowed with $\Delta \chi ^2 \sim 1$. In Fig. 21 we analyze the lifetime of the $\tilde{\tau }_{1}$. We see in the upper left panel of Fig. 21 that there is essentially no $\chi ^2$ penalty for $10^{-9}$ s $\lesssim \tau _{{\tilde{\tau }}_1} \lesssim 10^{-2}$ s, with lifetimes ${\sim } 10^{-10}$ s and ${\lesssim } 10^3$ s allowed with $\Delta \chi ^2 \lesssim 1$. Distinguishing a separated-vertex signature at the LHC would be challenging for smaller values of $\tau _{{\tilde{\tau }}_1}$, and there would be significant disruption of the successful conventional Big Bang nucleosynthesis calculations for $\tau _{\tilde{\tau }_1} \gtrsim 10^3$ s [183–192].

The upper right plot of Fig. 21 compares the $\tilde{\tau }_{1}$ lifetime with its mass. The plane is characterized by a strip with $800 \,\, \mathrm {GeV}\lesssim {{\tilde{\tau }}_1} \lesssim 1200 \,\, \mathrm {GeV}$ allowed at the 68% CL, while the 95% CL region is significantly wider, ranging from $m_{{\tilde{\tau }}_1} \sim 500 \,\, \mathrm {GeV}$ to $m_{{\tilde{\tau }}_1} \sim 2000 \,\, \mathrm {GeV}$.

The lower panels of Fig. 21 display the regions of the $(m_5, m_{1/2})$ (left) and $(m_{10}, m_{1/2})$ (right) planes in the SUSY SU(5) GUT model where the lowest-$\chi ^2$ points have $10^{-10} \, \mathrm{s}< \tau _{{\tilde{\tau }}_1} < 10^3 \, \mathrm{s}$. The colour-coding indicates the lifetimes of these points, as indicated in the legends. The contours for $\Delta \chi ^2 < 2.30 (5.99)$ relative to the best-fit point in our sample are shown as solid red and blue lines, respectively. One can see that larger lifetimes occur all over the displayed parameter space, with a slight preference for larger $m_5$ or $m_{10}$ values.

## Direct dark matter detection

As already mentioned, the PandaX-II experiment [83] has recently published results from its first 98.7 days of data, which currently provide the most stringent upper limits on the spin-independent DM scattering cross section on protons, $\sigma ^\mathrm{SI}_p$. In parallel, the LUX Collaboration [84] has presented preliminary constraints on $\sigma ^\mathrm{SI}_p$ from 332 days of data. We have combined these two constraints on $\sigma ^\mathrm{SI}_p$ into a single experimental likelihood function, which we have then convoluted with an estimate of the theoretical uncertainty in the calculation of $\sigma ^\mathrm{SI}_p$, as described in [16], to constrain the SUSY SU(5) GUT parameter space. This constraint has been used in obtaining the global fit whose results we have presented in the previous Sections. Here we discuss the future prospects for direct DM detection in light of our global fit.

**Fig. 22:**
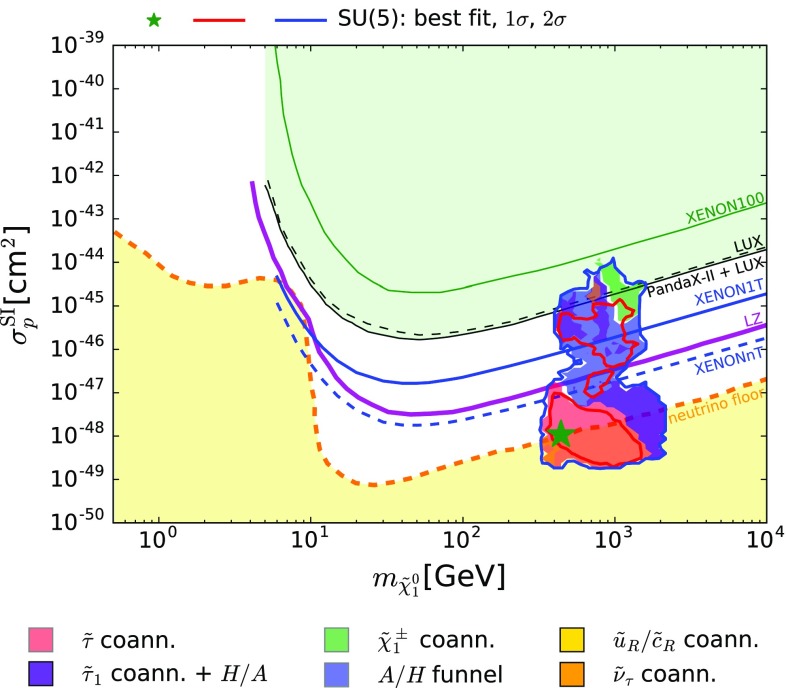
The $(m_{\tilde{\chi }^{0}_{1}}, \sigma ^\mathrm{SI}_p)$ plane in the SUSY SU(5) GUT model. The *solid green line* is the 95% CL upper limit from the XENON100 experiment, and the *dashed black solid line* is the new 95% CL upper limit from the LUX experiment. The *solid black line* shows the 95% CL exclusion contour for our combination of the PandaX-II and LUX experiments, the *solid purple line* shows the projected 95% exclusion sensitivity of the LUX-Zeplin (LZ) experiment, the *solid* and *dashed blue lines* show the projected 95% sensitivities of the XENON1T and XENONnT experiments, respectively, and the *dashed orange line* shows the astrophysical neutrino ‘floor’, below which astrophysical neutrino backgrounds dominate (*yellow region*). The other *line colours* and *shadings* within the 68 and 95% CL regions are the same as in Fig. 4

Figure 22 displays our results for the SUSY SU(5) GUT model in the $(m_{\tilde{\chi }^{0}_{1}}, \sigma ^\mathrm{SI}_p)$ plane. The combined PandaX-II/LUX constraint (black line) establishes a 95% CL that reaches $\sigma ^\mathrm{SI}_p$ $\simeq 2 \times 10^{-46}$ cm$^2$ for $m_{\tilde{\chi }^{0}_{1}} = 50 \,\, \mathrm {GeV}$ and ${\simeq } 10^{-45}$ cm$^2$ for $m_{\tilde{\chi }^{0}_{1}} = 500 \,\, \mathrm {GeV}$, providing the upper boundary of the 95% CL region in the $(m_{\tilde{\chi }^{0}_{1}}, \sigma ^\mathrm{SI}_p)$ plane seen in Fig. 22. We see that there are regions favoured at the 68% CL that lie relatively close to this boundary, whereas the main 68% CL region and the best-fit point have smaller values of $\sigma ^\mathrm{SI}_p$. We also note that the *H* / *A* funnel and $\tilde{\chi }^{\pm }_{1} - \tilde{\chi }^{0}_{1}$ DM mechanisms favour values of $\sigma ^\mathrm{SI}_p$ that are relatively close to the PandaX-II/LUX boundary, whereas the ${{\tilde{\tau }}_1} - \tilde{\chi }^{0}_{1}$ mechanism and its hybridization with the *H* / *A* funnel favour smaller values of $\sigma ^\mathrm{SI}_p$. The upcoming XENON1T [193] experiment will be able to probe the whole $\tilde{\chi }^{\pm }_{1}$ coannihilation region and a substantial part of the *H* / *A* funnel region.

We also display in Fig. 22 the projected 95% exclusion sensitivity of the future LUX-Zeplin (LZ) and XENONnT experiments (solid purple and dashed blue lines respectively) [193, 194], and the astrophysical neutrino ‘floor’ (dashed orange line) [195, 196], below which astrophysical neutrino backgrounds dominate (yellow region). We see that much of the ${{\tilde{\tau }}_1} - \tilde{\chi }^{0}_{1}$ coannihilation region and the region of its hybridization with the *H* / *A* funnel lie below the projected sensitivities of the LZ and XENONnT experiments, and substantial portions of them also lie below the neutrino ‘floor’. On the bright side, however, we recall that the ${{\tilde{\tau }}_1} - \tilde{\chi }^{0}_{1}$ region, in particular, lies at relatively small values of $m_5, m_{10}$ and $m_{1/2}$, offering greater prospects for detection at the LHC than, e.g., the $\tilde{\chi }^{\pm }_{1} - \tilde{\chi }^{0}_{1}$ region, so there is complementarity in the prospects of the LHC and direct DM experiments for probing the SUSY SU(5) GUT model, as was noted previously for other SUSY models [16].

## Summary and conclusions

We have explored in this paper the experimental, phenomenological, astrophysical and cosmological constraints on the minimal SUSY SU(5) GUT model. In this scenario the GUT-scale universal soft SUSY-breaking scalar mass $m_0$ is replaced by independent masses for the $\mathbf {10}$ and ${\bar{\mathbf{5}}}$ sfermions. This flexibility introduces some features that are novel compared to the GUT-universal CMSSM, NUHM1 and NUHM2.

In general we observe that many best-fit values of the coloured particles are within the reach of the HL-LHC, but that the preferred regions clearly extend beyond the reach of the final stage of the LHC. On the other hand, the best-fit masses of some electroweakly interacting particles are ${\sim } 500 \,\, \mathrm {GeV}$, offering the possibility of pair production at a collider with $\sqrt{s} \sim 1 \,\, \mathrm {TeV}$, as envisaged for the final stage of the ILC. Going to higher centre-of-mass energies, $\sqrt{s} \lesssim 3 \,\, \mathrm {TeV}$ as anticipated for CLIC, significant fractions of the 68% CL ranges of electroweak sparticle masses can be covered.

One novelty is the appearance of a ${\tilde{u}_R}/{\tilde{c}_R} - \tilde{\chi }^{0}_{1}$ coannihilation region that appears where $m_5^2$ is large and positive, $m_{10}^2$ is small and negative, and $m_{H_u}^2$ and $m_{H_d}^2$ are large and negative. On the other hand, we find that ${\tilde{t}_1} - \tilde{\chi }^{0}_{1}$ coannihilation is not important in the SUSY SU(5) GUT model, nor are the focus-point region and rapid $\tilde{\chi }^{0}_{1} \tilde{\chi }^{0}_{1}$ annihilation via direct-channel *h* and *Z* poles. We have checked that the ${\tilde{u}_R}/{\tilde{c}_R} - \tilde{\chi }^{0}_{1}$ coannihilation region is not yet excluded by searches for  events at the LHC, because the production rate is reduced compared to the case where all eight squarks are mass degenerate and the small ${\tilde{u}_R}/{\tilde{c}_R} - \tilde{\chi }^{0}_{1}$ mass difference suppresses this signature. However, this region may be accessible with future LHC runs.

We have also highlighted the possibility that a ${{\tilde{\nu }}_\tau }$ NLSP might have an important coannihilation role. Another novelty is the composition of the ${{\tilde{\tau }}_1}$ NLSP in a significant region of the model parameter space. In the GUT-universal CMSSM, NUHM1 and NUHM2 models, the universality of $m_0$ and the greater renormalization for SU(2) doublets impose a substantial mass difference between the ${{\tilde{\tau }}_2}$ and the ${{\tilde{\tau }}_1}$, with the latter being predominantly a ${{\tilde{\tau }}_R}$. However, in the SUSY SU(5) GUT model with $m_5 \ne m_{10}$, the ${\tilde{\tau }_R}$ and ${{\tilde{\tau }}_L}$ may have similar masses, and the off-diagonal entries in the ${{\tilde{\tau }}}$ mass matrix may cause large mixing and repulsion between the ${{\tilde{\tau }}_1}$ and ${{\tilde{\tau }}_2}$ masses.

On the other hand, one experimental signature that is shared by the SUSY SU(5) GUT model and GUT-universal models is the possible appearance of a long-lived (metastable) ${{\tilde{\tau }}_1}$. This is a feature of a significant fraction (but not all) of the ${\tilde{\tau }_1} -\tilde{\chi }^{0}_{1}$ coannihilation region.

The prospects for direct DM detection are mixed: they are relatively good in the $\tilde{\chi }^{\pm }_{1} - \tilde{\chi }^{0}_{1}$ coannihilation region, but less promising in the rapid *H* / *A* annihilation and hybrid regions, though potentially detectable in the planned LUX-Zeplin experiment. On the other hand, the ${{\tilde{\tau }}_1} -\tilde{\chi }^{0}_{1}$ coannihilation region probably lies beyond the reach of this experiment, as does part of the hybrid region. Indeed, portions of these regions lie below the neutrino ‘floor’. On the other hand, substantial parts of these regions are accessible to LHC searches for long-lived particles and .

## Footnotes

1 This possibility has also been noted in a supersymmetric SO(10) GUT framework [73].

2 We note that this problem becomes less severe for supersymmetry-breaking scales beyond a TeV [69].

3 We use a modified version of FeynHiggs 2.11.2 that includes two-loop QCD corrections in the evaluation of the ${\overline{\mathrm {DR}}}$ running top mass and an improved evalution of the top mass in the ${\overline{\mathrm {DR}}}$-on-shell conversion for the scalar tops.

4 An exception is provided by the ${\tilde{u}_R}$ and ${\tilde{c}_R}$, which may be much lighter than the gluino and other squarks in some regions of parameter space. We will discuss this possibility in detail below.

5 The $\tilde{u}_R/\tilde{c}_R- \tilde{\chi }^{0}_{1}$ coannihilation strip visible in the upper panels of Fig. 3 at $m_{\tilde{u}_R} \simeq m_{\tilde{\chi }^{0}_{1}} \sim 500 \,\, \mathrm {GeV}$ is the subject of a later dedicated discussion.

6 One could be concerned that summing up the $\chi ^2$ contributions from different simplified-model limits would overestimate the exclusion limit, since these signal regions are not necessarily independent. This would be indeed the case if the same event sample were confronted with multiple overlapping signal regions. In our case, however, the signal sample is divided into statistically independent sub-samples, corresponding to the simplified-model topologies $\tilde{g} \rightarrow b \bar{b} {\tilde{\chi }}_1^0, \rightarrow t \bar{t} {\tilde{\chi }}_1^0$, etc., and these sub-samples are confronted with the corresponding simplified-model limits only once. In such a case the  estimate (2) provides a conservative limit when there is no significant excess in the data.

7 A similar recasting method was used in [149]. See also [150] for another approach using simplified-model topologies.

8 A more conservative approach would be to choose the strongest search channel based on the expected sensitivity rather than that observed. However, the expected limit shown in [134] is similar to that observed, so we do not expect that this approach would lead to a significant change.

9 We note that the focus-point mechanism [163–168] does not play a role in the SU(5) model.

10 We have used Matplotlib [170] and PySLHA [171] to plot the results of our analysis.

11 The SLHA files for the best-fit point and other supplementary material can be found in [17].

12 An SLHA file corresponding to the ${\tilde{u}_R}/{\tilde{c}_R} - \tilde{\chi }^{0}_{1}$ coannihilation region can be found in [17].

13 The RGE evolution of the Yukawa couplings blows up for $\tan \beta \gtrsim 60$.

14 We note, however, that this constraint might weaken with a larger parameter sample.

15 The $\Delta \chi ^2$ functions for the NUHM2 subsample are calculated relative to its minimum $\chi ^2$, which is ${\sim } 0.4$ higher than the minimum $\chi ^2$ for the full SU(5) sample.

16 For conditions to have a long-lived $\tilde{\chi }^{\pm }_{1}$ with a bino-like LSP: see, e.g., [178].

17 The likelihood function for $h \rightarrow W W^*$ is very similar to that for $h \rightarrow Z Z^*$, because of custodial symmetry.
